# Characterization of *Smoc*-1 uncovers two transcript variants showing differential tissue and age specific expression in *Bubalus bubalis*

**DOI:** 10.1186/1471-2164-8-436

**Published:** 2007-11-28

**Authors:** Jyoti Srivastava, Sanjay Premi, Sudhir Kumar, Iqbal Parwez, Sher Ali

**Affiliations:** 1 Molecular Genetics Laboratory, National Institute of Immunology, Aruna Asaf Ali Marg, New Delhi-110 067, India

## Abstract

**Background:**

Secreted modular calcium binding protein-1 (*Smoc*-1) belongs to the BM-40 family which has been implicated with tissue remodeling, angiogenesis and bone mineralization. Besides its anticipated role in embryogenesis, *Smoc*-1 has been characterized only in a few mammalian species. We made use of the consensus sequence (5' CACCTCTCCACCTGCC 3') of 33.15 repeat loci to explore the buffalo transcriptome and uncovered the *Smoc*-1 transcript tagged with this repeat. The main objective of this study was to gain an insight into its structural and functional organization, and expressional status of *Smoc*-1 in water buffalo, *Bubalus bubalis*.

**Results:**

We cloned and characterized the buffalo *Smoc*-1, including its copy number status, *in-vitro *protein expression, tissue & age specific transcription/translation, chromosomal mapping and localization to the basement membrane zone. Buffalo *Smoc*-1 was found to encode a secreted matricellular glycoprotein containing two EF-hand calcium binding motifs homologous to that of BM-40/SPARC family. In buffalo, this single copy gene consisted of 12 exons and was mapped onto the acrocentric chromosome 11. Though this gene was found to be evolutionarily conserved, the buffalo *Smoc*-1 showed conspicuous nucleotide/amino acid changes altering its secondary structure compared to that in other mammals. *In silico *analysis of the *Smoc*-1 proposed its glycoprotein nature with a calcium dependent conformation. Further, we unveiled two transcript variants of this gene, varying in their 3'UTR lengths but both coding for identical protein(s). *Smoc*-1 evinced highest expression of both the variants in liver and modest to negligible in other tissues. The relative expression of variant-02 was markedly higher compared to that of variant-01 in all the tissues examined. Moreover, expression of *Smoc*-1, though modest during the early ages, was conspicuously enhanced after 1 year and remained consistently higher during the entire life span of buffalo with gradual increment in expression of variant-02. Immunohistochemically, *Smoc*-1 was localized in the basement membrane zones and extracellular matrices of various tissues.

**Conclusion:**

These data added to our understandings about the tissue, age and species specific functions of the *Smoc*-1. It also enabled us to demonstrate varying expression of the two transcript variants of *Smoc*-1 amongst different somatic tissues/gonads and ages, in spite of their identical coding frames. Pursuance of these variants for their roles in various disease phenotypes such as hepatocellular carcinoma and angiogenesis is envisaged to establish broader biological significance of this gene.

## Background

Matricellular or extracellular proteins do not contribute structurally to the extracellular milieu instead regulate the cell matrix interactions [1]. Basement membrane-40 (BM-40), SPARC (Secreted protein acidic and rich in cysteine) is an anti-adhesive secreted matricellular glycoprotein family [2,3] associated with tissue remodeling during normal developmental processes such as angiogenesis and bone mineralization [4]. Enhanced expression of SPARC has been reported in malignant tumors [5] and during early stages of embryogenesis but remain restricted in adult tissues [6]. The biological attributes of SPARC is to regulate the activities of collagen IV [7] platelet-derived growth factor (PDGF) [8,9] and vascular endothelial growth factor (VEGF) [10].

SPARC family proteins are characterized by the presence of a follistatin-like (FS) and a C-terminal extracellular (EC) calcium binding domains with two EF-hand binding motifs [11,12]. This family includes SC1/Hevin/QR1 [13,14], Testican [15], tsc36/Flik/FRP [16] and the recently described *SMOC*-2 [17] and *SMOC*-1 [18]. In addition to a calcium-binding EC domain, *SMOC*-1 consists of two thyroglobulin-like (TY1) domains, an FS domain and a novel *SMOC*-1 specific domain. *SMOC*-1 was localized within the basement membrane of various murine tissues and organs of different embryonic stages suggesting its significant role in embryonic development [18,19]. A perusal of literature, thus far, has not shown association of this gene with any satellites.

Satellite DNA represents a dynamic component of the eukaryotic genome [20,21]. The evolutionary conservation of a number of minisatellites either associated with non-coding or coding genes and their polymorphic status within/across the species suggest their vital regulatory roles in eukaryotic genomes [22-24]. However, association of minisatellites with the transcripts is thought to either regulate the transcription or bind proteins with diverse functional consequences [25]. In the earlier studies, we uncovered several transcripts representing known and novel genes from water buffalo using the Minisatellite Associated Sequence Amplification (MASA) approach and a consensus sequence of 33.15 repeat loci [26] originating from the human myoglobin gene [27]. Of these transcripts, one was found to represent the partial cDNA sequence of Secreted modular calcium binding protein-1 (*Smoc*-1), also known as SPARC related calcium binding protein-1.

Here, we describe isolation and characterization of full length *Smoc*-1 in water buffalo *Bubalus bubalis *including its domain organization, copy number status, *in silico *structural and functional analysis, *in-vitro *protein expression & purification, tissue & age specific transcription/translation and localization of the same onto the metaphase chromosomes & basement membrane zone. Biological significance of both the transcripts variants showing highest expression in liver is discussed.

## Results

### Characterization of buffalo *Smoc*-1

**Figure 1:**
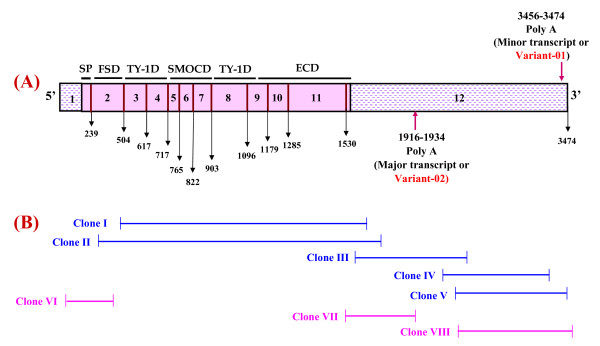
**Diagrammatic illustration showing cloning strategy of buffalo *Smoc*-1**. *Smoc*-1 structure representing 5'/3'UTRs, domain organization and nucleotide boundary of each exon is shown in (A). The strategy for isolation of the *Smoc*-1 is given in (B). Different fragments generated by end point PCR (blue) and RACE (pink) used to deduce the full length CDS are shown along with their nucleotide boundaries. Clone I covered nucleotides 318–1580; clone II, 119–1603; clone III, 1461–2473; clone IV, 2307–3328; and clone V, 2435–3428; clone VI, 1–649; clone VII, 1407–1915 and clone VIII, 2435–3474. Two transcript variants of *Smoc*-1 with their 3'UTR length variation are shown. Poly(A) tails for both the variants, -01 (3474 bp) and -02 (1934 bp) are marked by arrows in 'A'.

The cloning strategy for isolation of full length *Smoc*-1 CDS of 3474 bp and 1933 bp is demonstrated in Fig. 1A &1B. Clone I contained an insert of 1263 bp (PSmoc-1) lacking 5'/3' UTRs and signal peptide sequence [26]. Clone II (1414 bp) covered complete coding sequence from nucleotides 119–1603 but with partial 5' & 3' UTRs. The 3' UTR was covered by three fragments represented by clone III, IV & V of which clone III covered nucleotides from 1461–2473; clone IV, 2307–3328; and clone V, 2435–3428. The polyadenylation signals were accessed with 3'RACE followed by sequencing of 30 recombinants. This resulted in the identification of two other clones (Clone VII & VIII). Clone VII represented nucleotides 1407–1915 followed by 17 mer Poly(A) tail. Clone VIII covered 2435–3474 encompassing 18 mer Poly(A) tail. The 5' UTR represented by clone VI (nucleotides 1–649) was generated by 5' RACE. Following this strategy, full length CDS of *Smoc*-1 [FSmoc-1, GenBank: DQ159955 and EF446167] was deduced from different overlapping fragments (Fig. 1 & Additional file 1). The GC rich 5'-UTR of 239 bp was followed by an initiating ATG codon and the terminating TAA codon fell at nucleotide 1545. Thus, translation of the sequence from nucleotide 240 to 1544 encodes a putative protein of 435 amino acids with a calculated molecular mass of 48332 Da with a predicted oxidoreductase activity. Multiple sequence alignments showed that this protein is 95% and 98% identical to human & cattle *Smoc*-1, respectively (Table 1).

**Table 1:** Secreted modular calcium binding protein-1 from different species and their homology status with that of water buffalo *Bubalus bubalis*. The detailed information on Smoc-1 including accession numbers, gene length, exon numbers and chromosomal location are given.

**S.N.**	**Species**	**Accession numbers (Ensembl/NCBI)**	**Transcript length**	**Full length gene (In Kb)**	**No. of exons**	**Amino acid residues**	**Chromo-somal location**	**Homology with buffalo *Smoc*-1**		
								**CDS**	**Amino acids**	**Secondary structure**
1.	*Bubalus bubalis*	DQ159955	3274	NA	12	435	11	100%	100%	100%
2.	*Homo sapiens*	ENSG0000198732/AJ249900	3666	172.94	12	434	14q24.2	91%	95%	93%
3.	*Pan traglodytes*	ENSPTRT0000011881/XM_510036	3,669	154.09	14	435	14	90%	94%	94%
4.	*Bos taurus*	XM_612029	3473	NA	NA	434	10	98%	98%	97%
5.	*Mus musculus*	ENSMUSG0000021136/NM_022316	3472	179.58	13	463	12d3	84%	93%	89%
6.	*Rattus norvegicus*	ENSRNOG00000005998/NM_0102835	1359	193.42	12	452	6q24	85%	92%	89%
7.	*Canis familiaris*	ENSCAFT0000026288	1452	152.3	12	459	8	87%	95%	85%
8.	*Gallus gallus*	ENSGALG00000009415	1404	98.76	12	468	5	78%	84%	83%

### Buffalo *Smoc*-1 shows two transcript variants

**Figure 2:**
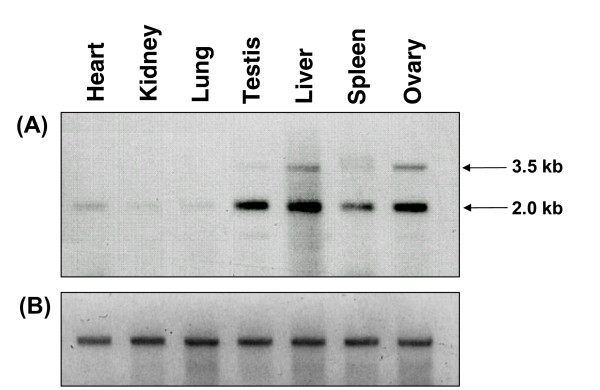
**Two transcript variants of *Smoc*-1**. Northern blot showing two transcript variants of *Smoc*-1 in different somatic and gonadal tissues of water buffalo. Note two distinct bands with varying intensity in each tissue along with highest expression in liver, and lowest in lung, kidney, and heart.

Northern blot detected two bands of 3.5 kb and 2.0 kb (Fig. 2) which were confirmed to be two transcript variants of *Smoc*-1 with RACE and sequencing (Fig. 1 and Additional file 1), variant-01 of 3474 bp [GenBank: DQ159955] and variant-02 of 1933 bp [GenBank: EF446167]. Both the variants encoded for identical proteins but showed differences in their 3'UTR length, polyadenylation signals and Poly(A) tails. In the 3' UTR of variant-01 & -02, five & two copies of mRNA instability motif (ATTTA), respectively, were observed. In addition, there were orthodox polyadenylation signals (AATAAA), 1787 and 334 bp downstream of the translation termination codon, in transcript variant-01 and -02, respectively (Additional file 1). Interestingly, two types of transcripts of *Smoc*-1 have been reported independently in the literature for human [GenBank: AJ249900 and BC011548] and cattle [GenBank: XM_612029 and NM_01079771]. Database search and multiple nucleotide sequence alignment of both the variants showed their conservation across various species (Additional file 2).

### Structure of the buffalo *Smoc*-1 and its phylogenetic delineation

The buffalo *Smoc*-1 has 12 exons, varying in length from 48 to 1916 bp in variant-01 and 48 to 402 bp in variant-02 (5^th ^being the smallest and 12^th^, the longest one) (Fig. 1 & Additional file 1). Each domain of *Smoc*-1 is encoded by one or more exons and the domain border coincide with the conserved splice sites. Buffalo *Smoc*-1 differed from other species in various aspects (Additional file 3), briefly, exon 8 was found to be more diverse at nucleotide level whereas exon 2 comprising the follistatin domain was most divergent at amino acid level. Exon 6 was the most conserved across the species. The buffalo *Smoc*-1 cross-hybridized to genomic DNA from 13 different species with almost equal signal intensity, confirming its faithful conservation across the species (Additional file 4A). Phylogenetic analysis demonstrated cattle as the closest species followed by human and chimpanzee while dog was the most distant one (Additional file 4B). In addition, the buffalo *Smoc*-1 also showed homology with other species such as birds, rodents and bony fishes (Additional file 4C). Details of the *Smoc*-1 gene(s) from different species along with their accession numbers are given in the Table 1.

### Domain Organization of *Smoc*-1

**Figure 3:**
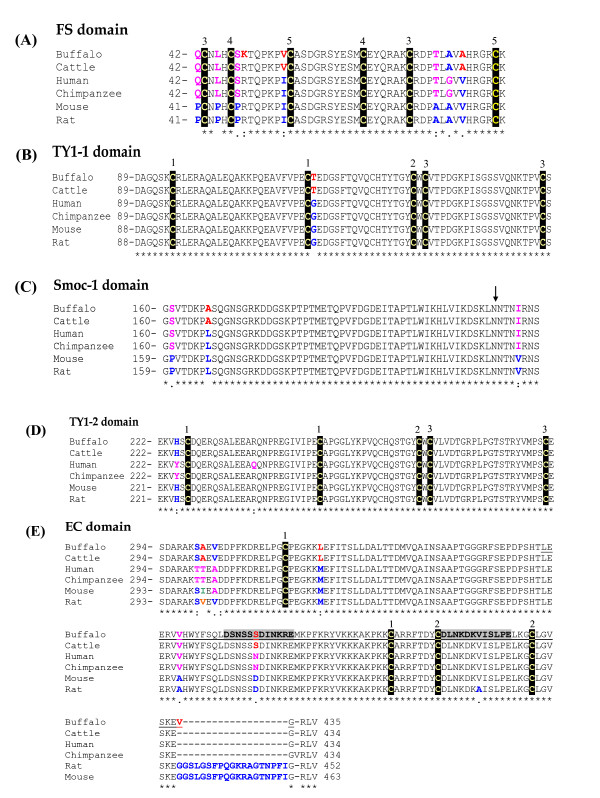
**Structure-based alignment of the Smoc-1 protein from different mammalian species**. The sequences were aligned across the species for FS (A), TY1-1 (B), *Smoc*-1 (C), TY1-2 (D) and EC (E) domains. Mutational hotspots in buffalo *Smoc*-1 are shown red boldface. Most of the observed changes in buffalo *Smoc*-1 were shared either by cattle or human. The "→" indicates the potential *N*-glycosylation site. Pairs of numbers above the sequence correspond to cysteines indicating the predicted disulphide bonds based on the disulphide linkage of BM-40 and thyroglobulin. Both EF hand motifs are underlined and calcium coordinating residues are overshadowed grey. Conserved amino acids are indicated by the stars below and cysteines with a black background.

Homology search for the buffalo Smoc-1 protein demonstrated presence of all the domains characteristic to the BM-40 family (Fig. 3). Accordingly, first 26 amino acids at the N-terminus conform well to the signal peptide consensus ending with a signal peptidase cleavage site [28]. Mature *Smoc*-1 comprises of 409 amino acids. Like in the human, all the essential features of each domain are conserved in buffalo. Since no transmembrane-spanning hydrophobic domain is present in the sequence, *Smoc*-1 is presumably secreted out from the cells. Further scrutiny allowed the distinction of five modules, an FS domain (Fig. 3A), a TY domain (Fig. 3B), a *Smoc*-1 unique domain (Fig. 3C), a second TY domain (Fig. 3D) and an EC domain (Fig. 3E). Residues 42–88 are homologous to the canonical FS domain, composed of two sub-domains with the second being similar to the Kazal domain. Structure-based alignment showed that all the six cysteines and the features of secondary structure are conserved in both the TY domains of buffalo *Smoc*-1. Further, two TY domains (residues 89–159 and 222–293) are separated by 62 amino acids unique to the *Smoc*-1. Detailed *in-silico *analysis unveiled a potential *N*-glycosylation site at Asn-214 and five O-glycosylation sites at Thr-155, -82, -184, -187 and -345 in buffalo *Smoc*-1. The C-terminus is homologous to the characteristic amphipathic α-helix and the helix-loop-helix motifs of the ECD of BM-40. However, based on the *in-silico *analyses, in contrast to the ECD of BM-40, both EF-hand motifs of *Smoc*-1 were found canonical indicating the calcium binding sites (Fig. 3E). Compared to other species, buffalo *Smoc*-1 showed some specific alteration like R49K in FS domain and V431 insertion in ECD. Some of these changes were from polar to non-polar amino acids and vice versa, similar to that in mouse/rat. In case of human/chimpanzee, these changes always maintained similar biochemical nature (Fig. 3). Interesting enough, predicted secondary structure showed alterations at the N-terminus involving replacement of 8 alpha-helices by equal number of beta-sheets and insertion of 3 helices in FS domain in comparison to other mammals (Additional file 5).

### Single copy of the *Smoc*-1 gene located on chromosome 11 in buffalo

**Figure 4:**
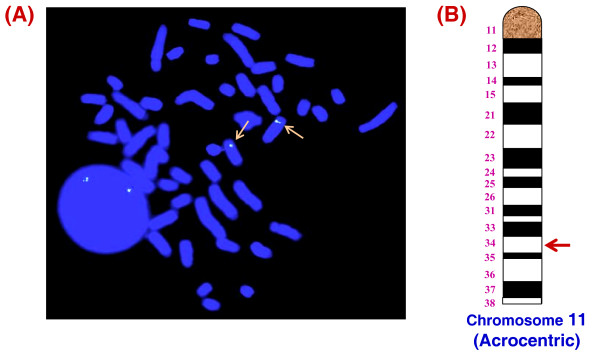
**Chromosomal mapping of *Smoc*-1 gene at chromosome 11 in buffalo**. Fluorescence *in situ *hybridization demonstrating the presence of *Smoc*-1 gene on the distal arm of the acrocentric chromosome 11 **(A) **and detailed mapping of this gene with respect to its position on the G-banded ideogram following the chromosome nomenclature standardized by ISCNDB, 2000 **(B)**.

For copy number status, using SYBR green assay and Real Time PCR, a straight curve was obtained with a slope -3.2 using 10 fold dilution series of buffalo blood/semen genomic DNA and F*Smoc*-1 plasmid as template. Ct increase of 3.3 per dilution and a single dissociation peak indicated maximum efficiency and high specificity of the primer sets. Extrapolation of this standard curve demonstrated the single copy status of the *Smoc*-1 per haploid genome in buffalo (Additional file 6). Chromosomal mapping of the same was also performed using Fluorescent in *situ *hybridization (FISH) which confirmed the localization of *Smoc*-1 gene on the distal end of the acrocentric chromosome 11 in buffalo (Fig. 4).

### Recombinant expression of *Smoc*-1

Affinity-purified recombinant *Smoc*-1 expressed in *E. coli BL2*1(*DE3*) revealed a major band at ~70 kDa in 10% SDS-PAGE under reduced conditions. The deduced molecular mass of mature *Smoc*-1 is 45.4 kDa and remaining ~25 kDa represent GST tag. The Anti-P*Smoc*1-pAb recognized native protein of ~70 kDa in the western-blot analysis. Anti-Sy*Smoc*-1-pAb generated against commercially synthesized 26 amino acids (69S to 95G) also showed the similar result (Additional file 7), substantiating its high specificity. The pre-immune serum did not detect any protein in the western blot (not shown).

### Highest expression of *Smoc*-1 transcript variants in liver

**Figure 5:**
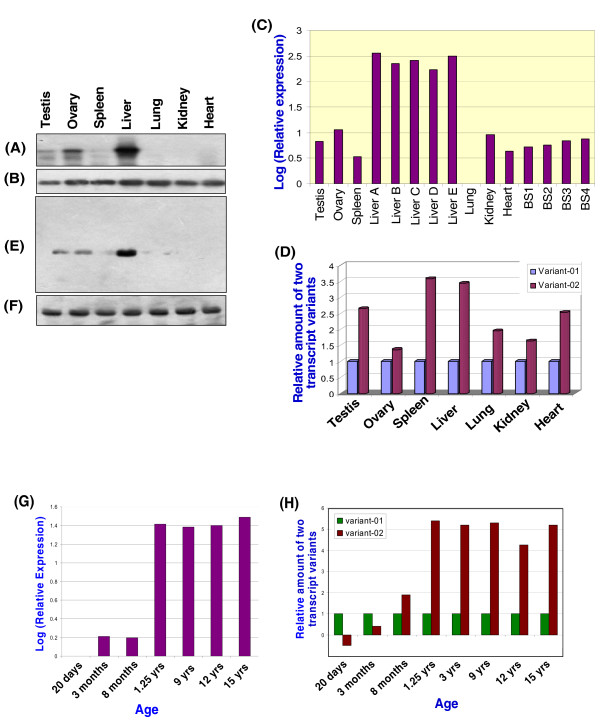
**Highest expression of *Smoc*-1 in liver**. RT-PCR showed expression in liver only but Southern hybridization detected reduced signals in testis, ovary and spleen (A) whereas control RT-PCR with β-actin showed almost equal intensity signal in each tissue (B). Quantitative expression of *Smoc*-1 based on Real Time PCR confirmed maximum expression (163–364 folds) in liver in five different animals compared to that in lung used as the calibrator (C). Note the buffalo spermatozoa from four different animals (BS1-4) also showed 4–7 folds transcripts in four different animals, similar to that in testis. Relative quantitation also demonstrated higher expression (1.2–3.5 times) of variant-02 compared to that of -01 in each of the tissues examined (D). Western blotting with anti-Sy*Smoc*1-pAb (E) and anti-β-actin-mAb as positive control (F) substantiated the highest expression of *Smoc*-1 protein in liver. The quantitative expression carried out using cDNA isolated from blood lymphocytes of different age group of animals is shown in (G). Note the markedly increased expression of *Smoc*-1 in animals 10 months and beyond. Relative quantitation showing higher expression of variant-01 in the animal up to ~6 months of age and that of variant-02 in the animals of age 6 months and beyond (H).

Northern blot analysis showed abundant *Smoc*-1 transcripts in liver and faint signals in testis and ovary. After prolonged exposure, the spleen, lung, kidney and heart also showed negligible to faint signals (Fig. 2). Similarly, RT-PCR followed by Southern hybridization detected reduced signals in spleen (Fig. 5A &5B) and negligible ones in lung, kidney and heart after prolonged exposure (not shown). Using quantitative expressional analysis, β-actin as an internal control and lung cDNA as calibrator, the highest level of expression of transcript variant-01 (165–364 folds) and -02 (360–697 folds) was observed in liver (Fig. 5C). This was substantiated further by expression data from the five additional animals. In the same assay, sperm cDNA showed similar level as that of testis. However, relatively higher amount (1.2–3.5 folds) of variant-02 was observed in all the tissues examined as compared to that of variant-01 (Fig. 5D). Based on these observations, the variant-02 may be addressed as "major transcript" and -01 as "minor" one.

Since all the mRNA transcripts may not translate into protein, the relative quantitation mentioned above was substantiated by the Western blot analysis using total tissue proteins and anti-Sy*Smoc*1-pAb which detected the ~45 kDa bands (mature protein). Interestingly, the strongest signal was detected in liver and faint ones in testis, ovary and spleen (Fig. 5E) whereas lung, kidney and heart were found to be devoid of *Smoc*-1 protein corroborating the RT-PCR and relative expressional analysis. As a control, anti-β-actin-mAb showed almost equal signal intensity in each tissue (Fig. 5F).

### Age specific progression of expression of *Smoc*-1 in water buffalo

We report for the first time, expression profile of *Smoc*-1 in water buffaloes of varying ages starting from 20 days to 15 years. Lowest expression of *Smoc*-1 was detected in the blood lymphocytes of animals aged 20 days with gradual increase in the expression (1.5–2 times) from 1 month-10 months. However, a sharp enhancement in the expression (25–30 times) was detected at the age of 1–1.25 years and this remained consistent up to the age of 15 years and beyond (Fig. 5G). The dramatic increase in expression of *Smoc*-1 at around 1 year of age was further confirmed by western blotting using anti-Sy*Smoc*1-pAb and total protein isolated from blood samples of the same animals (not shown).

The comparative expression analysis of the two transcript variants revealed a gradual increase of the variant-02 compared to that of variant-01 with the progression of age (Fig. 5H). The expression of variant-01 was higher in animal aged up to ~6 months, after which the variant-02 expression starts increasing gradually during the ages 6 to 15 months and remained consistent thereafter.

### Association of *Smoc*-1 with basement membranes

**Figure 6:**
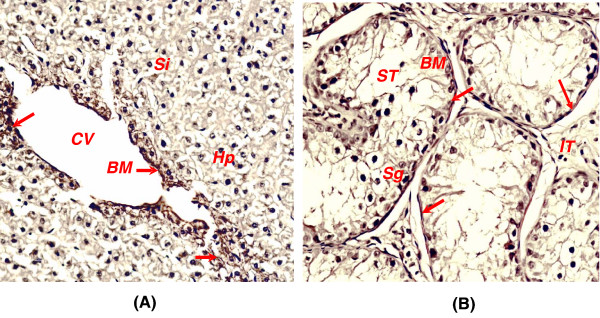
**Indirect Immunohistochemistry of buffalo tissue sections using Anti-SySmoc-1-pAb**. Distribution of Smoc-1 in basement membrane zone of endothelial cell layer and extracellular matrices of liver (**A**) and specific expression around basement membrane zone of the tubuli seminiferi in the testis (**B**). Note the localization of Smoc-1 protein in the basement membrane zones indicated by red arrows. The bars represent 5 μm in panels **A-B**. *Hp *denotes hepatocytes; *Si*, sinusoids; *CV*, central vein and *Em*, discontinuous endothelial cell of central vein in 'A', and *ST*, seminiferous tubules; *BM*, basement membrane zone; *Sg*, Spermatogonia and *IT*, interstitial tissues in '**B**'.

*Smoc*-1 was present abundantly in the basement membrane zone of discontinuous endothelial cell layer or the tunica media around the central veins in liver (Fig. 6A). In addition, *Smoc*-1 was found to be ubiquitously distributed in the connective tissues surrounding each lobule and extracellular matrices of space of Disse. In testis, *Smoc*-1 was abundant in the basement membrane zone surrounding coiled seminiferous tubules below the columnar epididymis and scarcely in the interstitial tissues (Fig. 6B). *Smoc*-1 was localized in the zona pellucida of ovary in buffalo (not shown) similar to that in mice. In other tissues also, it remained localized within the basement membrane zones.

## Discussion

Minisatellites have been implicated with gene regulation, chromosomal fragile sites and genome imprinting [29] but biological significance of their association with coding transcripts remains largely unresolved. Present study demonstrates the association of the consensus sequence of minisatellite 33.15 with the coding sequence of the *Smoc*-1 transcript [26]. However, the existing significance of this association remained unclear.

To date, *Smoc*-1 has been characterized only in a few mammals showing variations in domain organization. In other members of SPARC family, the FS domain is immediately followed by an EC domain [11] whereas in *Smoc*-1, the FS and EC domains are separated by the two TY domains which are themselves split by the novel *Smoc*-1 domain [18], thus maintaining its organizational uniqueness amongst SPARC family.

Anticipating the roles of orthologues separated millions of years ago has always been a difficult proposition, especially in the context of multidomain proteins with frequent insertions or deletions. Thus, biological functions for FS, TY and EC domains in *Smoc*-1 are still speculative. The FS domain is not only the characteristic of BM-40 family, but also found in other follistatin related genes like C6, C7, agrin, and the transmembrane receptors TMEFF1 and TMEFF2 [30,31]. Similarly, presence of TY domain in other proteins [32] makes it difficult to ascertain its function in the *Smoc*-1. The TY contain six cysteine residues including a characteristic CWCV tertrapeptide is also conserved in buffalo *Smoc*-1 [33]. The high content of aromatic amino acids in the unique *Smoc*-1 domain entails in formation of a folded domain with a hydrophobic core. Presence of two EF hand motifs in ECD of buffalo *Smoc*-1 is predicted for its calcium binding affinity as it has been confirmed experimentally using circular dichroism in human *SMOC*-1 [18]. Presence of acidic residues at positions 1,3,5,9 and 12, and the helix signatures encompassing the calcium binding loops are also conserved for both EF hand domains in buffalo *Smoc*-1.

Owing to >90% sequence homology with cattle, human and other species, buffalo *Smoc*-1 showed similar arrangement of various domains. Analysis of the gene structure in buffalo, human and mouse reveals intactness of each domain border [34] maintaining its reading frame even when some exon/intron is inserted or deleted. However, a number of specific alterations at nucleotide and amino acid levels were found to be unique to buffalo establishing their species specific organization. Two types of transcripts have been reported independently in GenBank for human and cattle *Smoc*-1 (Additional file 2) but their detailed characterization was lacking. In this study, we confirmed presence of two variants of this gene varying in their 3'UTR lengths. This may either be due to the presence of an alternative splice site within the possible inserted intron (12^th^) in the 3' region or an alternate splice site in the existing intron (11^th^) within the 3'UTR itself. However, first possibility seems to be invalid since end point PCR conducted with buffalo genomic DNA using primers from exon 11 and 12 gave rise to a single band of the similar size as that with cDNA. Further, analysis has shown that both the variants have polyadenylation signals followed by poly(A) tail upstream 30 and 16 nucleotides for variants -01 & -02, respectively. This is in agreement with the fact that the signals are most often present at 11–30 nucleotides upstream from the poly(A) tail [35]. However, presence of more copies of mRNA instability motifs in variant-01, involved in its degradation [36], compared to that in variant -02 supports relatively higher expression of the later.

Previous studies have shown that *Smoc*-1 mRNA is synthesized even during the early stages of mouse embryonic development. During the embryonic stage on day 12, and fetal stages days 14, 16, and 18, the protein is present in the basement membrane zones of various tissues like brain, skin, skeletal muscle, liver, kidney etc [19]. But, so far no report is available on the sustenance of expression of *Smoc*-1 during life-span of any of the species. Our work seems to be the first report showing a remarkable rise in the *Smoc*-1 expression during 10–14 months of age in buffaloes, followed by constant level maintained throughout the life span. As *Smoc*-1 is involved in cell-matrix interaction and bone mineralization, its fulminant expression at 10 months and beyond signifies its requirement for growth, development and possible sustenance of the animal.

Buffalo *Smoc*-1 is a single copy gene, presence of two variants of this gene may signify either for a backup of the transcripts if one is degraded/mutated or for the enhanced protein expression. In earlier studies, *Smoc*-1 mRNA was reported to be ubiquitously present in all the tissues of mice, showing abundance in ovary but negligible expression in liver and other tissues [19]. Contrary to this, buffalo liver was enriched with *Smoc*-1 transcripts as well as protein whereas other tissues contained fewer or no transcript/protein substantiating species and tissue specificity of this gene. Liver is primarily involved in vascular functions, metabolic regulation and secretory and excretory functions. Role of the other basement membrane proteins like agrin, collagen IV, laminin and fibronectin in liver cirrhosis and hepatocellular carcinoma has been studied [37] but no report is available on the functional attributes of *Smoc*-1 in liver. Buffalo may not be prone to hepatocellular carcinoma. However, since *Smoc*-1 is conserved across the species, it may not be inappropriate to study the expression of this gene in human hepatocellular carcinoma to ascertain its possible up- or down regulation. Owing to its involvement in cell proliferation, adhesion and tissue remodeling, *Smoc*-1 may also play a pivotal role in hepatocellular activities.

*Smoc*-1 has been localized in zona pellucida and extracellular matrix of mouse ovary. This was suggested to be crucial not only for survival of the oocyte but also for successful fertilization [18]. In this study, the *Smoc*-1 has been localized to the extracellular matrix and in the epithelial basement membrane zone of buffalo liver. In addition, staining around the seminiferous tubules and sertoli cells of testis substantiated the true basement membrane localization of *Smoc*-1 protein because the basal lamina of the seminiferous tubules in bovines is multilayered and possesses knob like protrusions [38].

The liver contains a unique extracellular matrix (ECM) within the space of Disse, which consists of basement membrane constituents as well as fibrillar ECM molecules. Though the basement membranes are mainly formed by a collagen IV, Laminin-1, and nidogen-1 network [39], the liver derived basement membrane also contains a unique isoform composition of type IV collagen, known to bind with the *Smoc*-1 protein [40]. Thus, *Smoc*-1 in ECM of buffalo liver seems to have an undisputed significance. Changes in the composition of ECM may be detrimental for the viability of hepatocytes during progression of liver cirrhosis. The role of SPARC/Osteonectin in human hepatocellular carcinoma has been reported [41]. Owing to its conservation in human and non-human systems, the fate of *Smoc*-1 gene may be studied in human liver cirrhosis, hepatocellular carcinoma and other liver infections to highlight its clinical aspects.

## Conclusion

In the present study, we demonstrated cloning, characterization and expressional analysis of the *Smoc*-1 in *Bubalus bubalis *for the first time and unveiled two transcript variants of this gene. Both the variants showed difference in their 3' UTR length but the deduced amino acid sequences were identical. Two EF-hand motifs in the ECD conformed well to its calcium binding affinity and N-glycosylation site at Asn-214 suggesting its glycoprotein nature. We also detected alterations in *Smoc*-1 at nucleotide, amino acid sequences and secondary structure levels amongst different species. Buffalo *Smoc*-1 transcript variants showed highest expression in liver demonstrating its tissue and species specific functions, in contrast to human and mouse where it expresses to the maximum in the ovary. The study also demonstrated the age specific expression of *Smoc*-1 intimating its role in postnatal development besides embryonic development. This study seems to be the first description of two transcript variants and tissue/age specific expression of the *Smoc*-1 in any mammalian species highlighting the possibilities of future research on its clinical aspects in the context of human/animal health.

## Methods

### Sample collection and isolation of genomic DNA, total RNA and cDNA synthesis

Blood and tissue samples of both the sexes of water buffalo were collected from local slaughterhouse, Delhi following strictly the guidelines of Institute's Ethical and Biosafety Committee. Buffalo semen samples were collected from the local dairy farm. Details of the genomic DNA from different species used in this study for cross-hybridization have been given earlier [26,42]. Total RNA was isolated from all the tissues, semen and blood samples from different age group of animals using standard protocols [42]. The cDNA synthesis was conducted using a commercially available kit (ABI, California, USA) and confirmed by PCR amplification using a set of bubaline derived β-actin (forward 5'CAGATCATGTTCGAGACCTTCAA3' and reverse 5'GATGATCTTGATC TTCATTGTGCTG 3') primers.

### Minisatellite Associated Sequence Amplification (MASA) with consensus of 33.15 repeat loci

MASA was conducted using 16 nucleotide long oligo (5' CACCTCTCCACCTGCC 3') primer derived from the consensus sequence of 33.15 repeat loci and cDNA from different tissues. The experimental details of MASA have been described earlier [26]. MASA uncovered several bands and one was found to represent partial sequence of *Smoc*-1 gene (P*Smoc*1: GenBank accession no. AY947405), lacking 5'/3'UTR and signal peptide.

### Amplification of full length *Smoc*-1 CDS using end point PCR and RACE

Full length *Smoc*-1 CDS was isolated using four sets of primers designed from of 5' and 3' regions of the human and cattle *Smoc*-1 sequences (GenBank Accession nos. AJ249900 and XM_612029) respectively using primer3 output [43]. Details of the primer sequences, Tm and corresponding sizes of amplicons are given in the additional file 8. End point PCR was conducted to amplify 3'UTR using cattle derived primers (JS275-JS997). The 5' UTR and polyadenylation signal at 3'UTR were identified using 5' & 3'RACE kits (Invitrogen, USA) and *Smoc*1-specific primer J5UTR & JS977/JS997, respectively. All the PCR reactions were conducted using Vent Polymerase (NEB, USA) following standard protocols. The amplicons were analyzed by gel electrophoresis followed by cloning and sequencing. The sequences so obtained were assembled into full length *Smoc*-1 (F*Smoc*-1). To ascertain the possible insertion of intron 12^th ^in 3'UTR, the primers JS990-SA991 & JS996-997 were used on buffalo genomic DNA as template to conduct end point PCR.

### Cloning, sequencing, Secondary structure prediction and phylogenetic delineation

The PCR products were tagged with dTTP and cloned into *pGEM*T-Easy vector (Promega, USA). The sequences of all the fragments were analyzed using Blast Search [44], ClustalW [45] and Gene Runner software. Finally, derived full length transcripts were submitted in the GenBank (Accession nos. DQ159955 and EF446167). The secondary structure of predicted protein was ascertained using Phyre software [46]. The calcium binding affinity, and N-glycosylation and O-glycosylation sites were predicted using different bioinformatics tools. Based on the homology, the phylogenetic tree [45] was constructed using *Smoc*-1 transcript(s) from buffalo and other species (Table 1). Only those species showing significant homologies (85–100%) were taken into consideration for phylogenetic analysis.

### Cross hybridization of buffalo *Smoc*-1 gene across the species

Approximately, 200 ng of heat denatured genomic DNA from different species was briefly run on 0.8% agarose gel and transferred onto the nylon membrane. Hybridization was conducted with labeled P*Smoc*-1*and *F*Smoc*-1 *pr*obes following standard procedure [42].

### Metaphase chromosome preparation and Fluorescence *in situ *hybridization

Approximately, 400 μl of whole blood from normal buffaloes was cultured for chromosome preparation following standard protocols [47]. Cattle derived BAC clone (Ctg9.CH240-54I18) representing full length *Smoc*-1 gene was used as probe. The clone was labeled with Fluorescein-12-dUTP using Nick Translation Kit from Vysis, (IL, USA). Fluorescein was detected with biotinylated anti-fluorescein antibody and FITC-avidin DCS (Vector Labs) following prescribed methods [47]. Map position of *Smoc*-1 gene on the chromosome was carried out following the International System for Chromosome Nomenclature of Domestic Bovids (ISCNDB 2000).

### Northern blot, RT-PCR and Southern Blotting

For Northern blot analyses, 5–10 μg of total RNA resolved on the 1% agarose gel was transferred onto the nylon membrane (Amersham Biosciences, USA). Hybridizations were performed under the high stringent conditions using standard procedure [26,42]. F*Smoc*1 probe was labeled with [^32^P] α-dCTP using rediprime™ II kit (Amersham Pharmacia biotech, USA). The Northern blot results were confirmed by RT-PCR with the internal primers designed from F*Smoc*-1 (F 5'-GGTTTCTCATAAGTGACCGTGACC-3', R 5'-TGAGATGACCTTGTCC TTGTTCAG-3') and cDNA of different tissues as template using thermal profile, 95°C-1 min, 59°C-1 min, 72°C-1 min. The products were transferred onto the nylon membrane followed by hybridization with [^32^P] α-dCTP labeled F*Smoc*1 using standard procedures. Bubaline derived β-actin gene probe and bacterial genomic DNA were used as positive and negative controls, respectively.

### Copy number calculation and quantitative expression using Real Time PCR

Copy number of *Smoc*-1 gene was calculated based on absolute quantitation using SYBR green assay and Sequence Detection System-7000 (SDS-7000, ABI, USA). Two primer pairs (Additional file 8) specific to 11^th ^exon of *Smoc*-1 were designed using Primer Express Software V2.0 (ABI, USA). The relative transcription of *Smoc*-1 across the tissues was assessed using the same primer set and equal quantity of total cDNA from different tissues and semen samples of buffalo. The relative expression of both the transcript variants was studied using three primer sets, two for both the variants (JSR1015-1018) and one specific for variant-01 (JSR1033-1034). Age specific expression was also carried out using cDNA from blood lymphocytes with the primer sets (JSR1015-1018) which picked up both the variants and another one JSR1033-1034 specific for variant-01. The total cDNA amounts from different tissues were optimized using a set of buffalo β-actin primers (forward 5'TCACGGAGCGTGGCTAC AG3' and reverse 5'TTGATGTCACGGACGATTTCC 3'). Presence of genomic DNA in the cDNA template was ruled out by using mRNA as template in several independent Real Time PCR and end point PCR reactions. Each reaction was repeated three times in triplicates. The details of the copy number calculation of the *Smoc-1 *mRNA transcripts have been described earlier [26,48].

### Protein Expression and Production of Anti-Smoc-1 Antiserum

Using P*Smoc*-1 as template, the *Smoc*-1 was re-amplified to accommodate a *Bam*HI site at the 5' end (5'-CGGGATCCCACCTCTCCACCTGCCCCAGG-3') and *Xho*I site at the 3' end (5'-CCCTCGAGTTAGACGAGGCGTCCTACTTC-3'). The resulting amplicon was cloned in pGEX-4T1 vector (Novagen, USA) at *BamH*I/*Xho*I sites. Expression of the recombinant GST-tag-*Smoc*1 in BL21 (DE3) was induced with 1 mM IPTG at 37°C for 4 h and the recombinant *Smoc*-1 protein was purified using GST-tag purification resin (Clontech, USA). A rabbit was immunized with purified recombinant pGEX-4T1-P*Smoc*1 using alum as an adjuvant to obtain the Anti-P*Smoc*-1-pAb. To ensure the specificity, primary antiserum (Anti-Sy*Smoc*1-pAb) was obtained for a commercially synthesized 26 amino acid (69S to 95G) long peptide, conjugated to Keyhole limpet hemocyanin (KLH), specific to *Smoc*-1 domain.

### Isolation of total protein from different tissues and Western Blotting

Denaturing 10% polyacrylamide gels were used under reducing conditions for analyzing culture medium and *E. coli *expressed proteins. Following electrophoresis, proteins were transferred onto nitrocellulose membranes. After blocking with 5% non-fat milk, 1%BSA in PBS for 45 min at room temperature, the membrane was probed with primary antibodies (anti-P*Smoc*1-pAb raised against pGEX-4T1-P*Smoc*1 anti-Sy*Smoc*1-pAb against synthesized peptide of *Smoc*-1). Secondary detection was carried out with goat anti-rabbit IgG conjugated with HRP (Bio-rad, USA) following standard protocol [49].

Total protein was isolated from different tissues using Tri-X reagent (MRC) followed by acetone precipitation. Protein quantity was normalized on 12% SDS PAGE followed by its transfer, hybridization and detection using standard procedure [49].

### Immunohistochemistry on Buffalo Tissue Sections

The distribution of Smoc-1 protein in different tissues was studied on paraffin sections by indirect Immunohistochemistry using Anti-SySmoc1-pAb (generated against synthesized polypeptide for Smoc-1 as stated earlier). Freshly prepared buffalo tissues were fixed for 1 hr in 4% paraformaldehyde/PBS and after dehydration, were embedded in paraffin. Sections were sliced and processed according to standard procedures [49]. After blocking with 1% BSA/TBS, the sections were incubated with the anti-P*Smoc*1-pAb followed by HRP-labeled goat anti-rabbit IgG (Bio-rad, USA) and detected using Di-amino Benzene (DAB) as substrate [49]. The sections were observed under BX-51 microscope (Olympus, JAPAN).

## List of Abbreviations

*Smoc*-1 Secreted modular calcium binding protein-1

BM-40 Basement membrane-40

SPARC Secreted protein acidic and rich in cysteines

UTR Untranslated region

PDGF Platelet derived growth factor

VEGF Vascular endothelial growth factor

TY Thyroglobulin domain

CDS cDNA sequence

RACE Random amplification of cDNA ends

ECD Extracellular domain

SDS-PAGE Sodium dodecyl sulphate-polyacrylamide gel electrophoresis

ECM Extracellular matrix

MASA Minisatellite associated sequence amplification

PCR Polymerase chain reaction

ISCNDB International System for Chromosome Nomenclature of Domestic Bovids

KLH Keyhole limpet hemocyanin

HRP Horse reddish peroxidase

RT-PCR Reverse transcriptase-Polymerase chain reaction

## Authors' contributions

JS: AB, JY, MT, ES

SP: AB, ES

SK: ES

IP: ES

SA: ES, FG

All the authors have checked the paper and have approved its publication in 'BMC Genomics'.

## Supplementary Material

Additional file 1 **Complete cDNA sequence with deduced amino acid sequences**. Complete cDNA sequence with the deduced amino acid sequences. The exons are shown in alternate colors and the start/stop codons in boldface & violet. The putative signal peptide sequence is underlined. The RNA instability motifs (ATTTA) are overshadowed yellow whereas polyadenylation signals (AATAAA) are underlined and shadowed yellow. The Poly(A) tail for transcript variant-02 is indicated by an inserted arrow and for variant-01 in boldface at the end of the sequence. Note the sequence 5'AAAAAA3' in transcript variant-01 is replaced by 5'AATAAA3' in variant-02 as evident by sequence analysis of 25 recombinant clones. Click here for file

Additional file 2 **Multiple sequence alignment for both transcript variants of *Smoc*-1 from buffalo, cattle and human**. Multiple nucleotide sequence alignment for both transcript variants from buffalo, cattle and human. In these species, variant-02 is shorter and almost of the same size due to possible conserved splice site in the 3'UTR. Polyadenylation signals (bold face) and Poly(A) tails (bold face and blue) are conserved in each variant in all the species. The nucleotides unique to buffalo are in blue and overshadowed grey. The changes specific to buffalo and cattle are shown in red and the ones similar to human are green. Click here for file

Additional file 3 **Multiple sequence alignment of *Smoc*-1 from different mammals**. Multiple nucleotide sequence alignment of *Smoc*-1 from different mammals. Some alterations were specific to buffalo or cattle (red) and many were either similar to human/chimpanzee (Pink) or to mouse/rat (Blue). Note > 90% nucleotide sequence conservation across the mammalian species. Click here for file

Additional file 4 **Evolutionary conservation of *Smoc*-1 across the species**. Cross hybridization of buffalo *Smoc*-1 with genomic DNA from different species (A), Phylogenetic tree based on sequence alignment of *Smoc*-1 gene(s) from different species (B) and neighbor joining tree based on BLAST result showing homology across the species with their accession numbers (C). Note that this gene is phylogentically conserved across the species. Click here for file

Additional file 5 **Secondary structure of *Smoc*-1 protein from different species**. Predicted secondary structures of *Smoc*-1 protein in different species. The replacement of helix formed by 8 residues with the beta-sheets, insertion of helices and the minor alterations throughout the protein are shown in red. Changes similar to cattle or human and the ones similar to rat/mouse or chimpanzee are shown in red and blue, respectively. The additional coils and helices in mouse and rat are due to bigger coding frame of *Smoc*-1 in these species. Click here for file

Additional file 6 **Copy number calculation of *Smoc*-1 gene**. Real Time PCR amplification plot based on ten fold dilution series of F*Smoc*-1 recombinant plasmid (A). Genomic DNA from blood of male/female buffalo and semen samples used as template (A) to obtain a standard curve using SYBR Green assay (B) which detected the single copy status of this gene. The value of R2, slope and intercept are given in the standard curve. Click here for file

Additional file 7 **Western blot using anti-*Smoc*-1 antibodies**. Anti-P*Smoc*1-pAb specifically generated against GST-*Smoc*1 recombinant protein showed ~70 kDa protein in western blotting (A). The same results were observed using Anti-Sy*Smoc*-1-pAb generated against the synthesized amino acids specific to *Smoc*-1 unique domain (B). TCL denotes total cell lysate; SS, sonicated supernatant; SP, sonicated pellet and EP, eluted protein. Click here for file

Additional file 8 **Details of primers used for analysis of *Smoc*-1**. List of primers used for amplification of full length *Smoc*-1*CDS*, Its relative expression and copy number calculation. The size of oligos, their annealing temperature and corresponding product size of the respective clones have been given in the table. Click here for file
