# Supplementary material for: Characterization of Smoc-1 uncovers two transcript variants showing differential tissue and age specific expression in Bubalus bubalis
Source: BMC Genomics. 2007 Nov 28;8:436. doi: 10.1186/1471-2164-8-436 (PMC2235864; doi:10.1186/1471-2164-8-436)
Supplement: Additional file 1 — Complete cDNA sequence with deduced amino acid sequences. Complete cDNA sequence with the deduced amino acid sequences. The exons are shown in alternate colors and the start/stop codons in boldface & violet. The putative signal peptide sequence is underlined. The RNA instability motifs (ATTTA) are overshadowed yellow whereas polyadenylation signals (AATAAA) are underlined and shadowed yellow. The Poly(A) tail for transcript variant-02 is indicated by an inserted arrow and for variant-01 in boldface at the end of the sequence. Note the sequence 5'AAAAAA3' in transcript variant-01 is replaced by 5'AATAAA3' in variant-02 as evident by sequence analysis of 25 recombinant clones. [file 1471-2164-8-436-S1.pdf]

GGCCACGCGTCGCGGA
GAGCGCCGCGCGCAGAGCACTCGCTAGCGCTGAGCTCCGCTCTCGGGGCGGTTTCATG
AGCGCGCGCGTTCGGCTGCAAGCCTCCGCGAGCCCGCGCTGCCGCCGCCCGCCGTCGC
CAGGGTCCCCGGGGTGGGAAGGAAAGGCAGGAAGGCCGGCGGCCGTGCGCTCCGTG
ATGACTGTGTCCCCTGACCGCAGCCCTCTGCCCGGACCGGCCTGGCACCATGCTGCCC
---M--L--P-

GCGCGCTGCGCCGGCCTGCTCACGCCCCACTTGCTGCTGGTGTTAGTGCAGCTGTCC
-A--R--C--A--G--L--L--T--P--H--L--L--L--V--L--V--Q--L--S-

CCGGCTCACGACCACCGCACCACCGGCCCCAGGTTTCTCATAAGTGACCGTGACCCT
-P--A--H--D--H--R--T--T--G--P--R--F--L--I--S--D--R--D--P-

CAGTGCAACCTCCACTGCTCCAGGACTCAACCCAAACCTGTCTGCGCCTCCGACGGC
-Q--C--N--L--H--C--S--R--T--Q--P--K--P--V--C--A--S--D--G-

AGGTCCTACGAGTCCATGTGTGAGTACCAGCGAGCTAAGTGCCGAGACCCAACCCTG
-R--S--Y--E--S--M--C--E--Y--Q--R--A--K--C--R--D--P--T--L-

GCTGTGGCGCATCGAGGCAGATGCAAAGACGCTGGCCAGAGCAAGTGTCGCCTGGAG
-A--V--A--H--R--G--R--C--K--D--A--G--Q--S--K--C--R--L--E-

CGGGCTCAGGCCCTGGGGCAAGCCAAGAAGCCCCAGGAGGCGGTGTTTGTCCCGGAG
-R--A--Q--A--L--G--Q--A--K--K--P--Q--E--A--V--F--V--P--E-

TGCACCGAGGATGGCTCCTTTACCCAGGTGCAGTGCCATACTTACACCGGGTACTGC
-C--T--E--D--G--S--F--T--Q--V--Q--C--H--T--Y--T--G--Y--C-

TGGTGTGTCACCCCAGACGGGAAGCCCATCAGTGGCTCTTCTGTGCAGAATAAAACT
-W--C--V--T--P--D--G--K--P--I--S--G--S--S--V--Q--N--K--T-

CCTGTATGTTCAGGTTCGGTCACCGATAAGCCCGCGAGCCAGGGTAACTCAGGAAGG
-P--V--C--S--G--S--V--T--D--K--P--A--S--Q--G--N--S--G--R-

AAAGATGACGGGTCTAAGCCGACACCCACGATGGAGACCCAGCCGGTGTTCGATGGA
-K--D--D--G--S--K--P--T--P--T--M--E--T--Q--P--V--F--D--G-

Additional file 1

Contd/-

GACGAAATCACAGCTCCCACTCTCTGGATTAAGCACTTGGTAATCAAGGACTCCAAA
-D--E--I--T--A--P--T--L--W--I--K--H--L--V--I--K--D--S--K-

CTGAACAACACCAACATAAGAAATTCAGAGAAAGTTCACTCGTGTGACCAGGAGAGA
-L--N--N--T--N--I--R--N--S--E--K--V--H--S--C--D--Q--E--R-

CAGAGCGCCCTGGAAGAGGCCCGGCAGAACCCCCGCGAGGGCATTGTGATCCCCGAG
-Q--S--A--L--E--E--A--R--Q--N--P--R--E--G--I--V--I--P--E-

TGTGCTCCTGGGGGGCTCTATAAACCAGTGCAGTGCCACCAGTCCACTGGCTACTGC
-C--A--P--G--G--L--Y--K--P--V--Q--C--H--Q--S--T--G--Y--C-

TGGTGTGTGCTGGTGGACACTGGGCGTCCGCTGCCGGGGACCTCCACACGCTATGTG
-W--C--V--L--V--D--T —G--R--P--L--P--G--T--S--T--R--Y--V-

ATGCCCAGTTGTGAGAGTGATGCCAGGGCTAAGAGTGCGGAGGTGGAGGACCCCTTC
-M--P--S--C--E--S--D--A--R--A--K--S--A--E--V--E--D--P--F-

AAGGACAGGGAGCTGCCAGGCTGTCCAGAAGGGAAGAAACTGGAATTTATCACCAGC
-K--D--R--E--L--P--G--C--P--E--G--K--K--L--E--F--I--T--S-

CTTCTGGACGCCCTCACCACGGACATGGTGCAGGCCATTAACTCAGCAGCGCCCACT
-L--L--D--A--L--T--T--D--M--V--Q--A--I--N--S--A--A--P--T-

GGAGGTGGGAGGTTCTCGGAGCCAGACCCCAGCCACACCCTGGAGGAGCGCGTGGTG
-G--G--G--R--F--S--E--P--D--P--S--H--T--L--E--E--R--V--V-

CACTGGTATTTCAGCCAGCTGGACAGCAACAGCAGCAGCGACATCAACAAGCGCGAG
-H--W--Y--F--S--Q--L--D--S--N--S--S--S--D--I--N--K--R--E-

ATGAAGCCCTTCAAGCGCTATGTGAAGAAGAAAGCCAAGCCCAAGAAATGTGCCCGG
-M--K--P--F--K--R--Y--V--K--K--K--A--K--P--K--K--C--A--R-

CGTTTCACTGACTACTGTGACCTGAACAAGGACAAGGTCATCTCACTGCCCGAGCTG
-R--F--T--D--Y--C--D--L--N--K--D--K--V--I--S--L--P--E--L-

AAGGGCTGCCTGGGTGTTAGCAAAGAAGTAGGACGCCTCGTC**TAA**GGAGCAGAAAGC
-K--G--C--L--G--V--S--K--E--V--G--R--L—-V--*------------

Contd/-

Additional file 1

CAAAGGGCAGGTGGAGAGACCAGGGAGGCAGGATGGATCATCAGACAGCTAACCTTCG
ATGTTGCCATGGCCCAGCCACATCCCATGTAACATAAGTGGTGCCCATCGTGTTTGCA
CTTTTAATAACTCTTATTTGTGTGTTTTCTTTTTCGGCTTC**ATTTA**TAAACACTAGTA
TCTAATATCGCAGTGGGAAAAGGAAAGGGAAGAAAGACTGTTTATTCTCTTTTATTGT
TAAGTTTTTGAATCTGCTACTGACAACTTTTAGGGTTTGGAGGGCGGGAGGCTTTCTG
GGACTGAGAAGAAAGAG**ATTTA**TATACTGTT**AATAAA**TATATATGTAAATTGTATAGT
AAAAAAAAAAAAAAAAA(transcript variant-002)
TCTTTTGTA CAGGTGTTGGCATTGCTATCTGTTTATTCCCCTCCCTCTCCCTGCTCT
GAGCTGTGAGAGCTCCGGACACACAGCCCCACTCTCTAGAACCCAGGACTCCATCCCT
GGCCAGCCTGGATTCCACTGTGATCACAGTGCAGACTCCGTGGGTATCTTTTCTGGTG
GGAGGAAGGGGCCACCTTCTGCCGTGGCTGTCAGAGCGGCAAGTCACTTGGCGGTTGA
CCTTCTCAAGGGAGGGAGTGGACATTGCAGGACAATGGGAGTGGCCCCTGGAGGGAGG
CCGGTAGCCCTCACGAGTCCCATCCTCCAACGCCCATGTGGTCAGGCCATCCAGACCC
CCAGGTGGCCCAGACTCAGTGGGTACACAGTGTCATTGGCGCCCACTGAACAAATTGC
CCTAAGGATTTGCGTTAGGGTGCCTTGAAACATTTCCAGCTACGTTTAGCATCTACTC
CACGTAAAGCAGGAGAGGGGAGGCAGAGAAGAAAGACACCCCGCGGGACCTTGT**ATTT**
AGTAGTTAAATGTAATATCTGAGCAGTGGAGGTAGAAGCACAGAAGGCTTGTCCTGGT
GAGTCCAGTGCCCACCCAGGGCTTTTCACCTCTCACACACCCATGAATGAGGCTTCCT
GAGACAACGCCCAATGCCGAGGTCAGACTAGGCAGCTACTTCTGCAGTCCTCTTTCTC
CCCTCCTGTTCTCCAGGTCAGCGTAAGCCTGCGGGAAGAGTTGCATCCATCACCTTGT
TGGTCACTCAACCGTTT**ATTTA**TTTTTTGTTGTTAAACTCAGTACTGAGGTTCTTCCT
GTTTTCCTAAACTCTCTTATGGGCTTCCAGGCTTGAGGCCAGTTCCAGGGCCAAATTC
ATGTTGGGCCTGTTACTTCTGCATCCCTTGGAAGTGAGGACAGAATGGCCCAGCCATG
GGGAAATCCAGGCCTAGCTTCCCACAGGCG**ATTTA**CTGTGATTCCAACGTGGACAGCC
CAGCCTTCTGGTCATACCCCAGCTTCCTCTTGCCCGGGGTGGCAGGGGTGGGGGCATG
CCCATCTGACAGTCATCCAACAAAGGGTGCCGGGTGACACGGAGCCCTCCTTTCCATG
AGCAGCCAGAGCAGCAGGGAGGGAGGGTGGGCAGTTTTCCAGGATGGGCGCCTTTGTG
GGTTACTTTTGGAAATCTGGCCGCATCTCTGCATCTCTAATCCCATCCCATCCTCTGA
CTGGAGAAGGTTCTTGCTGTCCTAATGAAAGTCCCAGAGGTTGTGTCAGGGTGACTGG
AGACCCCATCCCAACATGGTAGGATGGAACAAGAGCCCTGGCCCATCAGTCTGGACCA
GAAAGCCCCGTGTGCTGGCTGGGTGGACTTTCTGGGAGACCTCAGCCTCCTTCCCTGC
CCTGAAGGAAGCGCCTCCATGAAGAAAGTTGGAATCTCCCTGGGACATCTTCTCTCTC
ACACACGTGTGGAGGCTGAGTTGTGTGGTTTTCCTTTGTGAGGAGGGAGGGAGACCGT
TTGTAGCTTGTTTTATAAAA**AATAAA**AAATGCGTAAACCTTG**AAAAAAAAAAAAAAAA**
AA

Additional file 1
