# Supplementary material for: Characterization of Smoc-1 uncovers two transcript variants showing differential tissue and age specific expression in Bubalus bubalis
Source: BMC Genomics. 2007 Nov 28;8:436. doi: 10.1186/1471-2164-8-436 (PMC2235864; doi:10.1186/1471-2164-8-436)
Supplement: Additional file 2 — Multiple sequence alignment for both transcript variants of Smoc-1 from buffalo, cattle and human. Multiple nucleotide sequence alignment for both transcript variants from buffalo, cattle and human. In these species, variant-02 is shorter and almost of the same size due to possible conserved splice site in the 3'UTR. Polyadenylation signals (bold face) and Poly(A) tails (bold face and blue) are conserved in each variant in all the species. The nucleotides unique to buffalo are in blue and overshadowed grey. The changes specific to buffalo and cattle are shown in red and the ones similar to human are green. [file 1471-2164-8-436-S2.pdf]

Additional file 2: Multiple sequence alignment for both transcript variants from buffalo, cattle and human

```
Buffalo-01      ------------------------GGCCACGCGTCGCGGAG--AGCGCCGCGCGCAGAGC 34
Buffalo-02      ------------------------GGCCACGCGTCGCGGAG--AGCGCCGCGCGCAGAGC 34
Cattle-01       -GGCATCCAACCTGCTGCCGCCGCGGCCCAGCCGAGCGGAG--AGCGCCGCGCGCAGAGC 57
Cattle-02       GGGCATCCAACCTGCTGCCGCCGCGGCCCAGCCGAGCGGAG--AGCGCCGCGCGCAGAGC 58
Human-01        ---------GCCTGCTGCCGCCTGGGCCCCGCCGAGCGGAGCTAGCGCCGCGCGCAGAGC 51
Human-02        ----------CCTGCTGCCGCCTGGGCCCCGCCGAGCGGAGCTAGCGCCGCGCGCAGAGC 50
                                        ****  **   ******  *****************

Buffalo-01      ACTCGCTAGCGCTG-AGCTCCGCTCTCGGGGCGGTTTCATGAGCGCGCGCGTTCGGCTGC 93
Buffalo-02      ACTCGCTAGCGCTG-AGCTCCGCTCTCGGGGCGGTTTCATGAGCGCGCGCGTTCGGCTGC 93
Cattle-01       ACACGCTAGCGCTC-AGCTCCGCTCTCGGGGCGGTT-CATGAGCGCGCGC-TTCGGCTGC 114
Cattle-02       ACACGCTAGCGCTC-AGCTCCGCTCTCGGGGCGGTT-CATGAGCGCGCGC-TTCGGCTGC 115
Human-01        ACACGCTCGCGCTCCAGCTCCCCTCCTGCG-CGGTT-CATGACTGTGTCC-CCTGACCGC 108
Human-02        ACACGCTCGCGCTCCAGCTCCCCTCCTGCG-CGGTT-CATGACTGTGTCC-CCTGACCGC 107
                ** **** *****  ****** ***  * * ***** *****  * *  *    * * **

Buffalo-01      AAGCCTCCGCGAGCCCGCGCTGCCG--CCGCCCGCCG-TCGCCAGGGTCCCCGGGGTGGG 150
Buffalo-02      AAGCCTCCGCGAGCCCGCGCTGCCG--CCGCCCGCCG-TCGCCAGGGTCCCCGGGGTGGG 150
Cattle-01       A-GCCTCCGCGATCCCGCGCTGCCG--CCGCCCGCCG-TCGCCAGGGTCCCCCGGGGTGG 170
Cattle-02       A-GCCTCCGCGATCCCGCGCTGCCG--CCGCCCGCCG-TCGCCAGGGTCCCCCGGGGTGG 171
Human-01        A-GCCTCTGCGAGCCCCCGCCGCAGGACCACGGCCCGCTCCCCGCCGCCGCGAGGGCCCC 167
Human-02        A-GCCTCTGCGAGCCCCCGCCGCAGGACCACGGCCCGCTCCCCGCCGCCGCGAGGGCCCC 166
                * ***** **** *** *** ** *  ** *   *** ** **   * * *  ***

Buffalo-01      AAGGAAAGGCAGGAAGGCCGGCGCGGCCGTGCGCTCCGTGATGACTGTGTCCCCTGACCG 210
Buffalo-02      AAGGAAAGGCAGGAAGGCCGGCGCGGCCGTGCGCTCCGTGATGACTGTGTCCCCTGACCG 210
Cattle-01       GAAGGAAGGCAGGAAGGCCGGCGCGGCCGTGCGCTCCGTGAAGCCCGCCACCCCTGCCCG 230
Cattle-02       GAAGGAAGGCAGGAAGGCCGGCGCGGCCGTGCGCTCCGTGAAGCCCGCCACCCCTGCCCG 231
Human-01        GAGCGAAGGAAGGAAGGGAGGCGCG-CTGTGCGCCCCGCGGAGCCCGCGAACCCCGCTCG 226
Human-02        GAGCGAAGGAAGGAAGGGAGGCGCG-CTGTGCGCCCCGCGGAGCCCGCGAACCCCGCTCG 225
                 *   **** *******  ****** * ****** *** *  * * *    *** *  **

Buffalo-01      CAGCCCTCTGCCCGGACCGGCCTGGCACCATGCTGCCCGCGCGCTGCGCCGGCCTGCTCA 270
Buffalo-02      CAGCCCTCTGCCCGGACCGGCCTGGCACCATGCTGCCCGCGCGCTGCGCCGGCCTGCTCA 270
Cattle-01       CAGCCCGCTGCCCGGACCGGCCTGGCACCATGCTGCCCGCGCGCTGCGCCCGCCTGCTCA 290
Cattle-02       CAGCCCGCTGCCCGGACCGGCCTGGCACCATGCTGCCCGCGCGCTGCGCCCGCCTGCTCA 291
Human-01        CTGCCGGCTGCCCAGCCTGGC-TGGCACCATGCTGCCCGCGCGCTGCGCCCGCCTGCTCA 285
Human-02        CTGCCGGCTGCCCAGCCTGGC-TGGCACCATGCTGCCCGCGCGCTGCGCCCGCCTGCTCA 284
                * ***  ****** * * *** **************************** *********

Buffalo-01      CGCCCCACTTGCTGCTGGTGTTAGTGCAGCTGTCCCCGGCTCACGACCACCGCACCACCG 330
Buffalo-02      CGCCCCACTTGCTGCTGGTGTTAGTGCAGCTGTCCCCGGCTCACGACCACCGCACCACCG 330
Cattle-01       CGCCCCACTTGCTGCTGGCGTTAGTGCAGCTGTCCCCGGCTCACGACCACCGCACCACCG 350
Cattle-02       CGCCCCACTTGCTGCTGGCGTTAGTGCAGCTGTCCCCGGCTCACGACCACCGCACCACCG 351
Human-01        CGCCCCACTTGCTGCTGGTGTTGGTGCAGCTGTCCCCTGCTCGCGGCCACCGCACCACAG 345
Human-02        CGCCCCACTTGCTGCTGGTGTTGGTGCAGCTGTCCCCTGCTCGCGGCCACCGCACCACAG 344
                ****************** *** ************** **** ** ************ *

Buffalo-01      GCCCCAGGTTTCTCATAAGTGACCGTGACCCTCAGTGCAACCTCCACTGCTCCAGGACTC 390
Buffalo-02      GCCCCAGGTTTCTCATAAGTGACCGTGACCCTCAGTGCAACCTCCACTGCTCCAGGACTC 390
Cattle-01       GCCCCAGGTTTCTCATAAGTGACCGTGACCCTCAGTGCAACCTCCACTGCTCCAGGACTC 410
Cattle-02       GCCCCAGGTTTCTCATAAGTGACCGTGACCCTCAGTGCAACCTCCACTGCTCCAGGACTC 411
Human-01        GCCCCAGGTTTCTAATAAGTGACCGTGACCCACAGTGCAACCTCCACTGCTCCAGGACTC 405
Human-02        GCCCCAGGTTTCTAATAAGTGACCGTGACCCACAGTGCAACCTCCACTGCTCCAGGACTC 404
                ************* ***************** ****************************

Buffalo-01      AACCCAAACCTGTCTGCGCCTCCGACGGCAGGTCCTACGAGTCCATGTGTGAGTACCAGC 450
Buffalo-02      AACCCAAACCTGTCTGCGCCTCCGACGGCAGGTCCTACGAGTCCATGTGTGAGTACCAGC 450
Cattle-01       AACCCAAACCTGTCTGCGCCTCCGACGGCAGGTCCTATGAGTCCATGTGTGAGTACCAGC 470
Cattle-02       AACCCAAACCTGTCTGCGCCTCCGACGGCAGGTCCTATGAGTCCATGTGTGAGTACCAGC 471
Human-01        AACCCAAACCCATCTGTGCCTCTGATGGCAGGTCCTACGAGTCCATGTGTGAGTACCAGC 465
Human-02        AACCCAAACCCATCTGTGCCTCTGATGGCAGGTCCTACGAGTCCATGTGTGAGTACCAGC 464
                **********  **** ***** ** *********** **********************

Buffalo-01      GAGCTAAGTGCCGAGACCCAACCCTGGCTGTGGCGCATCGAGGCAGATGCAAAGACGCTG 510
Buffalo-02      GAGCTAAGTGCCGAGACCCAACCCTGGCTGTGGCGCATCGAGGCAGATGCAAAGACGCTG 510
Cattle-01       GAGCCAAGTGCCGAGACCCAACCCTGGCTGTGGCGCATCGAGGCAGATGCAAAGACGCTG 530
```

```
Cattle-02          GAGCCAAGTGCCGAGACCCAACCCTGGCTGTGGCGCATCGAGGCAGATGCAAAGACGCTG 531
Human-01           GAGCCAAGTGCCGAGACCCGACCCTGGGCGTGGTGCATCGAGGTAGATGCAAAGATGCTG 525
Human-02           GAGCCAAGTGCCGAGACCCGACCCTGGGCGTGGTGCATCGAGGTAGATGCAAAGATGCTG 524
                   **** ************** *******  **** ********* *********** ****

Buffalo-01         GCCAGAGCAAGTGTCGCCTGGAGCGGGCTCAGGCCCTGGGGCAAGCCAAGAAGCCCCAGG 570
Buffalo-02         GCCAGAGCAAGTGTCGCCTGGAGCGGGCTCAGGCCCTGGGGCAAGCCAAGAAGCCCCAGG 570
Cattle-01          GCCAGAGCAAGTGTCGCCTGGAGCGGGCTCAGGCCCTGGAGCAAGCCAAGAAGCCCCAGG 590
Cattle-02          GCCAGAGCAAGTGTCGCCTGGAGCGGGCTCAGGCCCTGGAGCAAGCCAAGAAGCCCCAGG 591
Human-01           GCCAGAGCAAGTGTCGCCTGGAGCGGGCTCAAGCCCTGGAGCAAGCCAAGAAGCCTCAGG 585
Human-02           GCCAGAGCAAGTGTCGCCTGGAGCGGGCTCAAGCCCTGGAGCAAGCCAAGAAGCCTCAGG 584
                   ******************************* ******* *************** ****

Buffalo-01         AGGCGGTGTTTGTCCCGGAGTGCACCGAGGATGGCTCCTTTACCCAGGTGCAGTGCCATA 630
Buffalo-02         AGGCGGTGTTTGTCCCGGAGTGCACCGAGGATGGCTCCTTTACCCAGGTGCAGTGCCATA 630
Cattle-01          AGGCGGTGTTTGTCCCGGAGTGCACCGAGGATGGCTCCTTTACCCAGGTGCAGTGCCATA 650
Cattle-02          AGGCGGTGTTTGTCCCGGAGTGCACCGAGGATGGCTCCTTTACCCAGGTGCAGTGCCATA 651
Human-01           AAGCTGTGTTTGTCCCAGAGTGTGGCGAGGATGGCTCCTTTACCCAGGTGCAGTGCCATA 645
Human-02           AAGCTGTGTTTGTCCCAGAGTGTGGCGAGGATGGCTCCTTTACCCAGGTGCAGTGCCATA 644
                   * ** *********** *****   ***********************************

Buffalo-01         CTTACACCGGGTACTGCTGGTGTGTCACCCCAGACGGGAAGCCCATCAGTGGCTCTTCTG 690
Buffalo-02         CTTACACCGGGTACTGCTGGTGTGTCACCCCAGACGGGAAGCCCATCAGTGGCTCTTCTG 690
Cattle-01          CTTACACCGGGTACTGCTGGTGTGTCACCCCAGACGGGAAGCCCATCAGTGGCTCTTCTG 710
Cattle-02          CTTACACCGGGTACTGCTGGTGTGTCACCCCAGACGGGAAGCCCATCAGTGGCTCTTCTG 711
Human-01           CTTACACTGGGTACTGCTGGTGTGTCACCCCGGATGGGAAGCCCATCAGTGGCTCTTCTG 705
Human-02           CTTACACTGGGTACTGCTGGTGTGTCACCCCGGATGGGAAGCCCATCAGTGGCTCTTCTG 704
                   ******* *********************** ** *************************

Buffalo-01         TGCAGAATAAAACTCCTGTATGTTCAGGTTCGGTCACCGATAAGCCCGCGAGCCAGGGTA 750
Buffalo-02         TGCAGAATAAAACTCCTGTATGTTCAGGTTCGGTCACCGATAAGCCCGCGAGCCAGGGTA 750
Cattle-01          TGCAGAATAAAACTCCTGTATGTTCAGGTTCGGTCACCGATAAGCCTGCGAGCCAGGGTA 770
Cattle-02          TGCAGAATAAAACTCCTGTATGTTCAGGTTCGGTCACCGATAAGCCTGCGAGCCAGGGTA 771
Human-01           TGCAGAATAAAACTCCTGTATGTTCAGGTTCAGTCACCGACAAGCCCTTGAGCCAGGGTA 765
Human-02           TGCAGAATAAAACTCCTGTATGTTCAGGTTCAGTCACCGACAAGCCCTTGAGCCAGGGTA 764
                   ******************************* ******** *****   ***********

Buffalo-01         ACTCAGGAAGGAAAGATGACGGGTCTAAGCCGACACCCACGATGGAGACCCAGCCGGTGT 810
Buffalo-02         ACTCAGGAAGGAAAGATGACGGGTCTAAGCCGACACCCACGATGGAGACCCAGCCGGTGT 810
Cattle-01          ACTCAGGAAGGAAAGATGATGGGTCTAAGCCGACACCCACGATGGAGACCCAGCCGGTGT 830
Cattle-02          ACTCAGGAAGGAAAGATGATGGGTCTAAGCCGACACCCACGATGGAGACCCAGCCGGTGT 831
Human-01           ACTCAGGAAGGAAAGATGACGGGTCTAAGCCGACACCCACGATGGAGACCCAGCCGGTGT 825
Human-02           ACTCAGGAAGGAAAGATGACGGGTCTAAGCCGACACCCACGATGGAGACCCAGCCGGTGT 824
                   ******************* ****************************************

Buffalo-01         TCGATGGAGACGAAATCACAGCTCCCACTCTCTGGATTAAGCACTTGGTAATCAAGGACT 870
Buffalo-02         TCGATGGAGACGAAATCACAGCTCCCACTCTCTGGATTAAGCACTTGGTAATCAAGGACT 870
Cattle-01          TCGATGGAGACGAAATCACAGCTCCCACTCTCTGGATTAAGCACTTGGTAATCAAGGACT 890
Cattle-02          TCGATGGAGACGAAATCACAGCTCCCACTCTCTGGATTAAGCACTTGGTAATCAAGGACT 891
Human-01           TCGATGGAGATGAAATCACAGCCCCAACTCTATGGATTAAACACTTGGTGATCAAGGACT 885
Human-02           TCGATGGAGATGAAATCACAGCCCCAACTCTATGGATTAAACACTTGGTGATCAAGGACT 884
                   ********** *********** ** ***** ******** ******** **********

Buffalo-01         CCAAACTGAACAACACCAACATAAGAAATTCAGAGAAAGTTCACTCGTGTGACCAGGAGA 930
Buffalo-02         CCAAACTGAACAACACCAACATAAGAAATTCAGAGAAAGTTCACTCGTGTGACCAGGAGA 930
Cattle-01          CCAAACTGAACAACACCAACATAAGAAATTCAGAGAAAGTTCACTCGTGTGACCAGGAGA 950
Cattle-02          CCAAACTGAACAACACCAACATAAGAAATTCAGAGAAAGTTCACTCGTGTGACCAGGAGA 951
Human-01           CCAAACTGAACAACACCAACATAAGAAATTCAGAGAAAGTCTATTCGTGTGACCAGGAGA 945
Human-02           CCAAACTGAACAACACCAACATAAGAAATTCAGAGAAAGTCTATTCGTGTGACCAGGAGA 944
                   ****************************************  * ****************

Buffalo-01         GACAGAGCGCCCTGGAAGAGGCCCGGCAGAACCCCCGCGAGGGCATTGTGATCCCCGAGT 990
Buffalo-02         GACAGAGCGCCCTGGAAGAGGCCCGGCAGAACCCCCGCGAGGGCATTGTGATCCCCGAGT 990
Cattle-01          GACAGAGCGCCCTGGAAGAGGCCCGGCAGAACCCCCGCGAGGGCATTGTGATCCCTGAGT 1010
Cattle-02          GACAGAGCGCCCTGGAAGAGGCCCGGCAGAACCCCCGCGAGGGCATTGTGATCCCTGAGT 1011
Human-01           GGCAGAGTGCCCTGGAAGAGGCCCAGCAGAATCCCCGTGAGGGTATTGTCATCCCTGAAT 1005
Human-02           GGCAGAGTGCCCTGGAAGAGGCCCAGCAGAATCCCCGTGAGGGTATTGTCATCCCTGAAT 1004
                   * ***** **************** ****** ***** ***** ***** ***** ** *

Buffalo-01         GTGCTCCTGGGGGGCTCTATAAACCAGTGCAGTGCCACCAGTCCACTGGCTACTGCTGGT 1050
Buffalo-02         GTGCTCCTGGGGGGCTCTATAAACCAGTGCAGTGCCACCAGTCCACTGGCTACTGCTGGT 1050
```

```
Cattle-01         GTGCTCCTGGGGGACTCTATAAACCAGTGCAGTGCCACCAGTCCACTGGCTACTGCTGGT 1070
Cattle-02         GTGCTCCTGGGGGACTCTATAAACCAGTGCAGTGCCACCAGTCCACTGGCTACTGCTGGT 1071
Human-01          GTGCCCCTGGGGGACTCTATAAGCCAGTGCAATGCCACCAGTCCACTGGCTACTGCTGGT 1065
Human-02          GTGCCCCTGGGGGACTCTATAAGCCAGTGCAATGCCACCAGTCCACTGGCTACTGCTGGT 1064
                   **** ******** ******** ******** ****************************

Buffalo-01        GTGTGCTGGTGGACACTGGGCGTCCGCTGCCGGGGACCTCCACACGCTATGTGATGCCCA 1110
Buffalo-02        GTGTGCTGGTGGACACTGGGCGTCCGCTGCCGGGGACCTCCACACGCTATGTGATGCCCA 1110
Cattle-01         GTGTGCTGGTGGACACTGGGCGTCCGCTGCCGGGGACCTCCACACGCTACGTGATGCCCA 1130
Cattle-02         GTGTGCTGGTGGACACTGGGCGTCCGCTGCCGGGGACCTCCACACGCTACGTGATGCCCA 1131
Human-01          GTGTGCTGGTGGACACAGGGCGCCCGCTGCCTGGGACCTCCACACGCTACGTGATGCCCA 1125
Human-02          GTGTGCTGGTGGACACAGGGCGCCCGCTGCCTGGGACCTCCACACGCTACGTGATGCCCA 1124
                   **************** ***** ******** ***************** **********

Buffalo-01        GTTGTGAGAGTGATGCCAGGGCTAAGAGTGCGGAGGTGGAGGACCCCTTCAAGGACAGGG 1170
Buffalo-02        GTTGTGAGAGTGATGCCAGGGCTAAGAGTGCGGAGGTGGAGGACCCCTTCAAGGACAGGG 1170
Cattle-01         GTTGTGAGAGTGATGCCAGGGCTAAGAGTGCGGAGGTGGAGGACCCCTTCAAGGACAGGG 1190
Cattle-02         GTTGTGAGAGTGATGCCAGGGCTAAGAGTGCGGAGGTGGAGGACCCCTTCAAGGACAGGG 1191
Human-01          GTTGTGAGAGCGACGCCAGGGCCAAGACTACAGAGGCGGATGACCCCTTCAAGGACAGGG 1185
Human-02          GTTGTGAGAGCGACGCCAGGGCCAAGACTACAGAGGCGGATGACCCCTTCAAGGACAGGG 1184
                   ********** ** ******** **** * * **** *** *******************

Buffalo-01        AGCTGCCAGGCTGTCCAGAAGGGAAGAAACTGGAATTTATCACCAGCCTTCTGGACGCCC 1230
Buffalo-02        AGCTGCCAGGCTGTCCAGAAGGGAAGAAACTGGAATTTATCACCAGCCTTCTGGACGCCC 1230
Cattle-01         AGCTGCCAGGCTGTCCAGAAGGGAAGAAACTGGAATTTATCACCAGCCTACTGGACGCCC 1250
Cattle-02         AGCTGCCAGGCTGTCCAGAAGGGAAGAAACTGGAATTTATCACCAGCCTACTGGACGCCC 1251
Human-01          AGCTACCAGGCTGTCCAGAAGGGAAGAAAATGGAGTTTATCACCAGCCTACTGGATGCTC 1245
Human-02          AGCTACCAGGCTGTCCAGAAGGGAAGAAAATGGAGTTTATCACCAGCCTACTGGATGCTC 1244
                   **** ************************ **** ************** ***** ** *

Buffalo-01        TCACCACGGACATGGTGCAGGCCATTAACTCAGCAGCGCCCACTGGAGGTGGGAGGTTCT 1290
Buffalo-02        TCACCACGGACATGGTGCAGGCCATTAACTCAGCAGCGCCCACTGGAGGTGGGAGGTTCT 1290
Cattle-01         TCACCACTGACATGGTGCAGGCCATTAACTCAGCAGCGCCCACTGGAGGTGGGAGGTTCT 1310
Cattle-02         TCACCACTGACATGGTGCAGGCCATTAACTCAGCAGCGCCCACTGGAGGTGGGAGGTTCT 1311
Human-01          TCACCACTGACATGGTTCAGGCCATTAACTCAGCAGCGCCCACTGGAGGTGGGAGGTTCT 1305
Human-02          TCACCACTGACATGGTTCAGGCCATTAACTCAGCAGCGCCCACTGGAGGTGGGAGGTTCT 1304
                   ******* ******** *******************************************

Buffalo-01        CGGAGCCAGACCCCAGCCACACCCTGGAGGAGCGCGTGGTGCACTGGTATTTCAGCCAGC 1350
Buffalo-02        CGGAGCCAGACCCCAGCCACACCCTGGAGGAGCGCGTGGTGCACTGGTATTTCAGCCAGC 1350
Cattle-01         CGGAGCCAGACCCCAGCCACACCCTGGAGGAGCGCGTGGTGCACTGGTATTTCAGCCAGC 1370
Cattle-02         CGGAGCCAGACCCCAGCCACACCCTGGAGGAGCGCGTGGTGCACTGGTATTTCAGCCAGC 1371
Human-01          CAGAGCCAGACCCCAGCCACACCCTGGAGGAGCGGGTAGTGCACTGGTATTTCAGCCAGC 1365
Human-02          CAGAGCCAGACCCCAGCCACACCCTGGAGGAGCGGGTAGTGCACTGGTATTTCAGCCAGC 1364
                   * ******************************** ** **********************

Buffalo-01        TGGACAGCAACAGCAGCAGCGACATCAACAAGCGCGAGATGAAGCCCTTCAAGCGCTATG 1410
Buffalo-02        TGGACAGCAACAGCAGCAGCGACATCAACAAGCGCGAGATGAAGCCCTTCAAGCGCTATG 1410
Cattle-01         TGGACAGCAACAGCAGCAGCGACATCAACAAGCGCGAGATGAAGCCCTTCAAGCGCTACG 1430
Cattle-02         TGGACAGCAACAGCAGCAGCGACATCAACAAGCGCGAGATGAAGCCCTTCAAGCGCTACG 1431
Human-01          TGGACAGCAATAGCAGCAACGACATTAACAAGCGGGAGATGAAGCCCTTCAAGCGCTACG 1425
Human-02          TGGACAGCAATAGCAGCAACGACATTAACAAGCGGGAGATGAAGCCCTTCAAGCGCTACG 1424
                   ********** ******* ****** ******** *********************** *

Buffalo-01        TGAAGAAGAAAGCCAAGCCCAAGAAATGTGCCCGGCGTTTCACTGACTACTGTGACCTGA 1470
Buffalo-02        TGAAGAAGAAAGCCAAGCCCAAGAAATGTGCCCGGCGTTTCACTGACTACTGTGACCTGA 1470
Cattle-01         TAAAGAAGAAAGCCAAGCCCAAGAAATGTGCCCGGCGTTTCACTGACTACTGTGACCTGA 1490
Cattle-02         TAAAGAAGAAAGCCAAGCCCAAGAAATGTGCCCGGCGTTTCACTGACTACTGTGACCTGA 1491
Human-01          TGAAGAAGAAAGCCAAGCCCAAGAAATGTGCCCGGCGTTTCACCGACTACTGTGACCTGA 1485
Human-02          TGAAGAAGAAAGCCAAGCCCAAGAAATGTGCCCGGCGTTTCACCGACTACTGTGACCTGA 1484
                   * ***************************************** ****************

Buffalo-01        ACAAGGACAAGGTCATCTCACTGCCCGAGCTGAAGGGCTGCCTGGGTGTTAGCAAAGAAG 1530
Buffalo-02        ACAAGGACAAGGTCATCTCACTGCCCGAGCTGAAGGGCTGCCTGGGTGTTAGCAAAGAAG 1530
Cattle-01         ACAAGGACAAGGTCATCTCACTGCCCGAGCTGAAGGGCTGCCTGGGTGTTAGCAAAGAAG 1550
Cattle-02         ACAAGGACAAGGTCATCTCACTGCCCGAGCTGAAGGGCTGCCTGGGTGTTAGCAAAGAAG 1551
Human-01          ACAAAGACAAGGTCATTTCACTGCCTGAGCTGAAGGGCTGCCTGGGTGTTAGCAAAGAAG 1545
Human-02          ACAAAGACAAGGTCATTTCACTGCCTGAGCTGAAGGGCTGCCTGGGTGTTAGCAAAGAAG 1544
                   **** *********** ******** **********************************

Buffalo-01        TAGGACGCCTCGTCTAAGGAGCAGAAAGCCAAAGGGCAGGTGGAGAGACCAGGGAGGCAG 1590
```

```
Buffalo-02        TAGGACGCCTCGTCTAAGGAGCAGAAAGCCAAAGGGCAGGTGGAGAGACCAGGGAGGCAG 1590
Cattle-01         TAGGACGCCTCGTCTAAGGAGCAGAAAACCAAAGGGCAGGTGGAGAGACCAGGGAGGCAG 1610
Cattle-02         ---GACGCCTCGTCTAAGGAGCAGAAAACCAAAGGGCAGGTGGAGAGACCAGGGAGGCAG 1608
Human-01          ---GACGCCTCGTCTAAGGAGCAGAAAACCCAAGGGCAGGTGGAGAGTCCAGGGAGGCAG 1602
Human-02          TAGGACGCCTCGTCTAAGGAGCAGAAAACCCAAGGGCAGGTGGAGAGTCCAGGGAGGCAG 1604
                     ************************ ** **************** ************

Buffalo-01        GATGGATCATCAGACAGCTAACCTTCGATGTTGCC-ATGGCCCAGCCACATCCCATGTAA 1649
Buffalo-02        GATGGATCATCAGACAGCTAACCTTCGATGTTGCC-ATGGCCCAGCCACATCCCATGTAA 1649
Cattle-01         GATGGATCATCAGACAGCTAACCTTCGACGTTGCC-ACGGCCCAGCCACATCCCATGTAA 1669
Cattle-02         GATGGATCATCAGACAGCTAACCTTCGACGTTGCC-ACGGCCCAGCCACATCCCATGTAA 1667
Human-01          GATGGATCACCAGACACCTAACCTTCAGCGTTGCCCATGGCCCTGCCACATCCCGTGTAA 1662
Human-02          GATGGATCACCAGACACCTAACCTTCAGCGTTGCCCATGGCCCTGCCACATCCCGTGTAA 1664
                  ********* ****** *********   ****** * ***** ********** *****

Buffalo-01        CATAAGTGGTGCCCATCGTGTTTGCACTTTTAATAACTCTTATTTGTGTGTTTTCTTTTT 1709
Buffalo-02        CATAAGTGGTGCCCATCGTGTTTGCACTTTTAATAACTCTTATTTGTGTGTTTTCTTTTT 1709
Cattle-01         CATAAGTGGTGCCCATCGTGTTTGCACTTTTAATAACTCTTATTTGTGTGTTTTCTTTTT 1729
Cattle-02         CATAAGTGGTGCCCATCGTGTTTGCACTTTTAATAACTCATATTTGTGTGTTTTCTTTTT 1727
Human-01          CATAAGTGGTGCCCACCATGTTTGCACTTTTAATAACTCTTACTTGCGTGTTTTGTTTTT 1722
Human-02          CATAAGTGGTGCCCACCATGTTTGCACTTTTAATAACTCTTACTTGCGTGTTTTGTTTTT 1724
                  *************** * ********************* ** *** ******* *****

Buffalo-01        CGGCTTCATTTTTAAACACTAGTATCTAATATCGCAGTGGGAAAAGGAAAGGGAAGAAAG 1769
Buffalo-02        CGGCTTCATTTTTAAACACTAGTATCTAATATCGCAGTGGGAAAAGGAAAGGGAAGAAAG 1769
Cattle-01         CGGCTTCATTTTTAAACACCAGTATCTAATATTGCAGTGGGAAAAGGAAAGGGAAGAAAG 1789
Cattle-02         CGGCTTCATTTTTAAACACCAGTATCTAATATTGCAGTGGGAAAAGGAAAGGGAAGAAAG 1787
Human-01          -GGTTTCATTTTAAAACACCAATATCTAATACCACAGTGGGAAAAGGAAAGGGAAGAAAG 1781
Human-02          -GGTTTCATTTTAAAACACCAATATCTAATACCACAGTGGGAAAAGGAAAGGGAAGAAAG 1783
                   ** ******** ****** * *********   **************************

Buffalo-01        ACTGTTTATTCTCTTTTATTGTTAAGTTTTTGAATCTGCTACTGACAACTTTTAGGG--- 1826
Buffalo-02        ACTGTTTATTCTCTTTTATTGTTAAGTTTTTGAATCTGCTACTGACAACTTTTAGGG--- 1826
Cattle-01         ACT----ATTCTCTTTTATTGTTAAGTTTTTGGATCTGCTACTGACAACTTTTAGGG--- 1842
Cattle-02         ACT----ATTCTCTTTTATTGTTAAGTTTTTGGATCTGCTACTGACAACTTTTAGGG--- 1840
Human-01          ACTTTATTCTCTCTCTTATTGT-AAGTTTTTGGATCTGCTACTGACAACTTTTAGAGGGT 1840
Human-02          ACTTTATTCTCTCTCTTATTGT-AAGTTTTTGGATCTGCTACTGACAACTTTTAGAGGGT 1842
                  ***      ***** ******* ********* ********************** *

Buffalo-01        -TTTGGAGGGCGGGAG-----GCTTTCTGGGACTGAGAAGAAAGAGATTTATATACTGTA 1880
Buffalo-02        -TTTGGAGGGCGGGAG-----GCTTTCTGGGACTGAGAAGAAAGAGATTTATATACTGTA 1880
Cattle-01         -TTTGGAGGGCGGGGA-----GCTTTCTGGGACTGAGAAGAAAGAGATTTATATACTGTA 1896
Cattle-02         -TTTGGAGGGCGGGGA-----GCTTTCAGGGACTGAGAAGAAAGAGATTTATATACTGTA 1894
Human-01          TTTGGGGGGGTGGGGGAGGGTGTTGTTGGGGCCTGAGAAGAAAGAGATTTATATGCTGTA 1900
Human-02          TTTGGGGGGGTGGGGGAGGGTGTTGTTGGGGC-TGAGAAGAAAGAGATTTATATGCTGTA 1901
                   ** ** *** ***       * * *  ***  ********************* *****

Buffalo-01        TATAAATATATATGTAAATTGTATAGTTCTTTTGTACAGGTGTTGGCATTGCTATCTGTT 1940
Buffalo-02        TATAAATATATATGTAAATTGTATAGTTCTTTTGTAAAAAAAAAAAAAAAAAA------- 1933
Cattle-01         TATAAATATATATGTAAATTGTATAGTTCTTTTGTACAGGTGTTGGCATTGCTATCTGTT 1956
Cattle-02         TATAAATATATATGTAAATTGTAAATAAAAAAAAAAAAAA-------------------- 1934
Human-01          TATAAATATATATGTAAATTGTATAGTTCTTTTGTACAGGCATTGGCATTGCTGTTTGTT 1960
Human-02          TATAAATATATATGTAAATTGTATAGTTCTTTTGTACAGGCAAAAAAAAAAAAAAAAAA- 1960
                  *********************** *          * *

Buffalo-01        TATTCCCCTCCCTCTCCCTGCTCTGAGCTGTGAGAGCTCCGGACACACAGCCCCACTCTC 2000
Buffalo-02        ------------------------------------------------------------
Cattle-01         TATTCTCCTCCCTCTCCCTGCTCTGAGCTGTGAGAGCTCCGGACACACAGCCCCACTCTC 2016
Cattle-02         ------------------------------------------------------------
Human-01          TATTTCTCTCCCTCTGCCTGCTGTGGGTGGTGGGCACTCTGGACACATAGTCCAGCTTTC 2020
Human-02          ------------------------------------------------------------

Buffalo-01        TAGAACCCAGGACTCCATCCCTGGCCAGCCTGGATTCCA--------------------- 2039
Buffalo-02        ------------------------------------------------------------
Cattle-01         TAGAACCCAGGACTCTATCCCTGGCCAGCCTGAATTCCA--------------------- 2055
Cattle-02         ------------------------------------------------------------
Human-01          TAAAATCCAGGACTCTATCCTGGGCCTACTAAACTTCTGTTTGGAGACTGACCCTTGTGT 2080
Human-02          ------------------------------------------------------------
```

```
Buffalo-01        ---------------CTGTGATCACAGTGCAGACTCCGTGGGTATCTTTTCTGGTGGGAG 2084
Buffalo-02        ------------------------------------------------------------
Cattle-01         ---------------CTGTGATCACAGTGCAGACTCCATGGGTATCTTTTCTGGTGGGAG 2100
Cattle-02         ------------------------------------------------------------
Human-01          ATAAAGACGGGAGTCCTGCAATTGTACTGCGGACTCCACGAGT-TCTTTTCTGGTGGGAG 2139
Human-02          ------------------------------------------------------------

Buffalo-01        GAAGGGGCCACCTTCTGCCGT-GGCTGTCAGAGC-GGCAAGTCACTTGGCGGTTGACCTT 2142
Buffalo-02        ------------------------------------------------------------
Cattle-01         GAAGGGACCACCTTCTGCCGT-GGCTGTCAGAGC-GGCAAGTCACTTGGCGGTTGACCTT 2158
Cattle-02         ------------------------------------------------------------
Human-01          GACTATATTGCCCCATGCCATTAGTTGTCAAAATTGATAAGTCACTTGGCTCTCGGCCTT 2199
Human-02          ------------------------------------------------------------

Buffalo-01        CTCAAGGGAGG--GAGTGGACATTGCAGGACAATGGGAGTGGCCCCTGGAGGGAGGCCGG 2200
Buffalo-02        ------------------------------------------------------------
Cattle-01         CTCAAGGGAGG--GAGTGGACATTGCAGGACAATGGGAGTGGCCCCTGGAGGGAGGCCTG 2216
Cattle-02         ------------------------------------------------------------
Human-01          GTCCAGGGAGGTTGGGCTAAGGAGAGATGGAAACTGCCCTGGGAGAGGAAGGGAGTCCAG 2259
Human-02          ------------------------------------------------------------

Buffalo-01        ----------TAGCCCTCACGAGTCCCATCCTCCAACG--CCCATGTGGTCAGGCCATCC 2248
Buffalo-02        ------------------------------------------------------------
Cattle-01         ----------TAGCCCTCACAAGTCCCATCCTCCAACG--CCCATGTGGTCAGGCCATCC 2264
Cattle-02         ------------------------------------------------------------
Human-01          ATCCCATGAATAGCCCACACAGGTACCGGCTCTCAGAGGGTCCGTGCATTCCTGCTCTCC 2319
Human-02          ------------------------------------------------------------

Buffalo-01        AGACCCCCAGGTGGCCCAGACTCAGTGGGTACAC-AGTGTCATTGGCGCCCACTGAACAA 2307
Buffalo-02        ------------------------------------------------------------
Cattle-01         AGACCCCCAGGTGGCCCAGACTCAGTGGGTGCA---GTGTTGTTGGCGCCCGCTGAACAA 2321
Cattle-02         ------------------------------------------------------------
Human-01          GGACCCCCAAAGGGCCCAGCATTGGTGGGTGCACCAGTATCTTAGTGACCCTCGGAGCAA 2379
Human-02          ------------------------------------------------------------

Buffalo-01        ATTGCCCT--AAGGATTTGCGTTAGGGTGCCTTGAAACATTTCCAGCTACGTTTAGCATC 2365
Buffalo-02        ------------------------------------------------------------
Cattle-01         ATTGCCCT--AAGGATTTGCGTTAGGGTCCCTTGAAACATTTCCAGCTACGTTTAGCCTC 2379
Cattle-02         ------------------------------------------------------------
Human-01          ATTATCCACAAAGGATTTGCATTACG-TCACTCGAAACGTTTTCATCCATGCTTAGCATC 2438
Human-02          ------------------------------------------------------------

Buffalo-01        TACTCCACGTAAAGCAGGAGAGGGGAGGCAGAGAAGAAA--GACACCCCGCGGGACCTTG 2423
Buffalo-02        ------------------------------------------------------------
Cattle-01         TACTCCACGTAAAGCAGGAGAGGGGAGGCAGAGAAGAAAAAGACACCCCGCGGGACCTTT 2439
Cattle-02         ------------------------------------------------------------
Human-01          TACTCTGTATAACGCATGAGAGGGGAGGCAAAGAAGAAAAGGACACACGGAAGGGCCTTT 2498
Human-02          ------------------------------------------------------------

Buffalo-01        TATTTAGTAGTTAAATGTAATATCTGAGCAGTGGAGGTAGAAGCACAGAAGGCTTGTCCT 2483
Buffalo-02        ------------------------------------------------------------
Cattle-01         TATTTAGTAGTTAAATGTAATATCTGAGCAGTGGAGGTAGAAGCACAGAAGGCCTGTCCT 2499
Cattle-02         ------------------------------------------------------------
Human-01          AAAAAAGTAGATA--TTTAATATCTAAGCAGGGGAGGGGACAGGACAGAAAGCCTGCACT 2556
Human-02          ------------------------------------------------------------

Buffalo-01        GGTGAGTCCAGTGCCCACCCAGGG-CTTTTCACCTCTC-ACACACCCATGAATGAGGCTT 2541
Buffalo-02        ------------------------------------------------------------
Cattle-01         GGTGAGTCCAGTGCCCACCCAAGG-CTTCTCACCTGTC-ACACACCCATGAATGAGGCTT 2557
Cattle-02         ------------------------------------------------------------
Human-01          GAGGGGTGCGGTGCCAACAGGGAAACTCTTCACCTCCCTGCAAACCTACCAGTGAGGCTC 2616
Human-02          ------------------------------------------------------------
```

```
Buffalo-01        CCTGAGACAACGCC----CAATGCCGAGGTCAGACTAGGCAGCTACTTCTGCAGTCCTCT 2597
Buffalo-02        ------------------------------------------------------------
Cattle-01         CCTGAGACAGCGCC----CAGTGCCAAGGTCAGACTAGGCAGCTACTTCTGCAGTCCTCT 2613
Cattle-02         ------------------------------------------------------------
Human-01          CCAGAGACGCAGCTGTCTCAGTGCCCAGGGGCAGATTGGGTGTGACCTCTCCACTCCTCC 2676
Human-02          ------------------------------------------------------------

Buffalo-01        TTCTCCC-CTCCTGTTCTCCAGGTCAGCGTAAGCCTGCG-GGAAGAGTTG-------CAT 2648
Buffalo-02        ------------------------------------------------------------
Cattle-01         TTCTCCC-CTCCTGTTCTCCAGCTCAGCGTAAGCCTGTG-GGAAGAGTTG-------TGT 2664
Cattle-02         ------------------------------------------------------------
Human-01          ATCTCCTGCTGTTGTCCTAGTGGCTATCACAGGCCTGGGTGGGTGGGTTGGGGGAAGTGT 2736
Human-02          ------------------------------------------------------------

Buffalo-01        CCATCACCTTGTTGGTCACTCAACCGTTTATTTTTTTTTTGTTGTTAAACTCAGTACTGA 2708
Buffalo-02        ------------------------------------------------------------
Cattle-01         CCATCACCTTGTTGGTCATTCAACCTTTTCTATTTTTTTCTTT-TTAAACTCAGTACCGA 2723
Cattle-02         ------------------------------------------------------------
Human-01          CAGTCACCTTGTTGGTAACACTAAAGTTGTTTTGTTGGTTTTTTAAAAACCCAATACTGA 2796
Human-02          ------------------------------------------------------------

Buffalo-01        GGTTCTTCCTGTTTTCCTAAACTCTCTTATGGGCTTCCAGGCTTGAGGCCAGTTCCAGG- 2767
Buffalo-02        ------------------------------------------------------------
Cattle-01         GGTTCTTCCTGTTTTCCTAAACGCTCTTATGGGCTTCCAGGCTTGAGGCCAGTTCCAGG- 2782
Cattle-02         ------------------------------------------------------------
Human-01          GGTTCTTCCTGTTCCCTCAAGTTTTCTTATGGGCTTCCAGGCTTTAAGCTAATTCCAGAA 2856
Human-02          ------------------------------------------------------------

Buffalo-01        GCCAAATTCATGTTGGGCCTGTTACTTCTGCATCCCTTGGAAGTGAGGACAGAATGGCCC 2827
Buffalo-02        ------------------------------------------------------------
Cattle-01         GCCAAATTCATGTTGGGCCTGTTACTTCTGCATCCCTTGGAAGTGAGGACGGAATGGCCC 2842
Cattle-02         ------------------------------------------------------------
Human-01          GTAAAACTGATCTTGGGTTTCCTA-TTCTGCCTCCCCTAGAAGGGCAGGGGTGATAACCC 2915
Human-02          ------------------------------------------------------------

Buffalo-01        AGCCATGGGGAAATCCAGGCCTAGCTTCCCACAGGCGTT---------TTACTGTGATTC 2878
Buffalo-02        ------------------------------------------------------------
Cattle-01         AGCCATGGGGAAATCCAGGCCTAGCTTCCCACAGGCGTT---------TTACTGTGATTC 2893
Cattle-02         ------------------------------------------------------------
Human-01          AGCTACAGGGAAATCCCGGCCCAGCTTTCCACAGGCATCACAGGCATCTTCCGCGGATTC 2975
Human-02          ------------------------------------------------------------

Buffalo-01        CAACGTGGACAGCCCAGCCTTCTGGTCATACCCCAGCTTCCTCTTGCCCGGGGTGGCAGG 2938
Buffalo-02        ------------------------------------------------------------
Cattle-01         CAACGTGGACCGCCCAGCCTTCTGGTCATACCCCAGCTTCCTCTTGCCCGGGGTGGCAGG 2953
Cattle-02         ------------------------------------------------------------
Human-01          TAGGGTGGGCTGCCCAGCCTTCTGGTCTGAGGCGCAGCTCCCTCTGCCCAGGT------- 3028
Human-02          ------------------------------------------------------------

Buffalo-01        GGTGGGGGCATGCCCATCTGACAGTCATCCAACAAAGGGTGCCGGGTGACACGGAGCCCT 2998
Buffalo-02        ------------------------------------------------------------
Cattle-01         GGTGGGGGCATGCCCATCCGACAGTCATCCAACAAAGGGTGCCGGGTGACACCGAGCCCT 3013
Cattle-02         ------------------------------------------------------------
Human-01          ------GCTGTGCCTATTCAAGTGGCCTTCAG-GCAGAGCAGCAAGTGGCCCTTAGCGCC 3081
Human-02          ------------------------------------------------------------

Buffalo-01        CCTTTCCATGAGCAGCCAGAGCAGCAGGGAGGGAGGGTGGGCAGTTTTCCAGGATGGGCG 3058
Buffalo-02        ------------------------------------------------------------
Cattle-01         CCTTTCCGTGAGCAGCCAGAGCGGCAGGGAGGGAGGGTGGGCAGTTTTCCAGGACCGGCG 3073
Cattle-02         ------------------------------------------------------------
Human-01          CCTTCCCATAAGCAGCTGTGGTGGCAGTGAGGGAGGTTGGGTAGC--CCTGGACTGGTCC 3139
Human-02          ------------------------------------------------------------
```

```
Buffalo-01       CCTTTGTGGGTTACTTTTGGAAATCTGGCCGCATCT----------CTG--CATCTCTAA 3106
Buffalo-02       ------------------------------------------------------------
Cattle-01        CCTTTGTGGATTACTTTTGGAAATCTGGCCGCATCT----------CTGTGCATCTCCTA 3123
Cattle-02        ------------------------------------------------------------
Human-01         CCTCCTCAGATCACCCTTGCAAATCTGGCCTCATCTTGTATTCCAACCCGACATCCCTAA 3199
Human-02         ------------------------------------------------------------

Buffalo-01       ------TCCCATCCCATCC----------------------------------------- 3119
Buffalo-02       ------------------------------------------------------------
Cattle-01        ------CCCCATCCCATCC----------------------------------------- 3136
Cattle-02        ------------------------------------------------------------
Human-01         AAGTACCTCCACCCGTTCCGGGTCTGGAAGGCGTTGGCACCACAAGCACTGTCCCTGTGG 3259
Human-02         ------------------------------------------------------------

Buffalo-01       ---------------------------------------TCTGACTGGAGAAGGTTCTTG 3140
Buffalo-02       ------------------------------------------------------------
Cattle-01        ---------------------------------------TCTGACTGGAGAAGGTTCTTG 3157
Cattle-02        ------------------------------------------------------------
Human-01         GAGGAGCACAACCTTCTCGGGACAGGATCTGATGGGGTCTTGGGCTAAAGGAGGTCCCTG 3319
Human-02         ------------------------------------------------------------

Buffalo-01       CTGTCCTAATGAAAGTCCCAGAGGTTGTGTCAGGG-TGACTGGAGACCCCATCCCAACAT 3199
Buffalo-02       ------------------------------------------------------------
Cattle-01        CTGTCCTAATGAAAGTCCCAGAGGTTGTGTCAGGG-TGACTGGAGACCCCATCCCAACAT 3216
Cattle-02        ------------------------------------------------------------
Human-01         CTGTCCTGGAGAAAGTCCTAGAGGTTATCTCAGGAATGACTGGTGGCCCTGCCCCAACGT 3379
Human-02         ------------------------------------------------------------

Buffalo-01       GGTAGGATGGAACAAGAGCCCTGGCCCATCAGTCTGGACCAGAAAGCCCCGTGTGCTGG- 3258
Buffalo-02       ------------------------------------------------------------
Cattle-01        GGTAGGATGGAACGAGAGCCCTGGCCCATCAGTCTGGACCAGAAAGCCCCGTGTGCTGG- 3275
Cattle-02        ------------------------------------------------------------
Human-01         GGAAAGGTGGGAAGGAAGCCTTCTCCCATTAGCCCCAATGAGAGAACTCAACGTGCCGGA 3439
Human-02         ------------------------------------------------------------

Buffalo-01       -CTGGGTGGACTTTCTGGGAGACCTCAGCCTCCTTCCCTGCCCTGAAGGAAGC------- 3310
Buffalo-02       ------------------------------------------------------------
Cattle-01        -CTGGGTGGACTTTCTGGGAGACCTCCGCCTCCTTCCCTGCCCTGAAGGAAGC------- 3327
Cattle-02        ------------------------------------------------------------
Human-01         GCTGAGTGGGCCTTGCACGAGACACTGGCCCCACTTTCAGGCCTGGAGGAAGCATGCACA 3499
Human-02         ------------------------------------------------------------

Buffalo-01       ------------GCCTCCATGAAGAAAGTTGGAATCTC-------CCTGGGACATCTTCT 3351
Buffalo-02       ------------------------------------------------------------
Cattle-01        ------------ACCTCCGTGAAGAAAGTTGGAATCTC-------CCTGGGACGTCTTCT 3368
Cattle-02        ------------------------------------------------------------
Human-01         CATGGAGACGGCGCCTGCCTGTAGATGTTTGGATCTTCGAGATCTCCCCAGGCATCTTGT 3559
Human-02         ------------------------------------------------------------

Buffalo-01       CTCTCACA----CACGTGTGGAGGCTGAGTTGTGTGGTTTTCCTTTGTGA-GGAGGGAGG 3406
Buffalo-02       ------------------------------------------------------------
Cattle-01        CTCTCACA----CACGTGTGGAGGCTGAGTTGTGTGGTTTTCCTTTGTGA-GGAGGGAGG 3423
Cattle-02        ------------------------------------------------------------
Human-01         CTCCCACAGGATCGTGTGTGTAGGTGGTGTTGTGTGGTTTTCCTTTGTGAAGGAGAGAGG 3619
Human-02         ------------------------------------------------------------

Buffalo-01       GAGACCGTTTGTAGCTTGTTTTATAAAAAAATAAAAAATGCGTAAACCTTG 3474
Buffalo-02       --------------------------------------------------
Cattle-01        GAGACCGTTTGTAGCTTGTTTTATAAAAAAATAAAAAATGCGTAAACCTTG 3473
Cattle-02        --------------------------------------------------
Human-01         GAAACTATTTGTAGCTTGTTTTATAAAAAAATAAAAAATGGGTAAATCTTG 3669
Human-02         --------------------------------------------------
```
