# Supplementary material for: Characterization of Smoc-1 uncovers two transcript variants showing differential tissue and age specific expression in Bubalus bubalis
Source: BMC Genomics. 2007 Nov 28;8:436. doi: 10.1186/1471-2164-8-436 (PMC2235864; doi:10.1186/1471-2164-8-436)
Supplement: Additional file 3 — Multiple sequence alignment of Smoc-1 from different mammals. Multiple nucleotide sequence alignment of Smoc-1 from different mammals. Some alterations were specific to buffalo or cattle (red) and many were either similar to human/chimpanzee (Pink) or to mouse/rat (Blue). Note > 90% nucleotide sequence conservation across the mammalian species. [file 1471-2164-8-436-S3.pdf]

Additional file 3: Multiple sequence alignment of *Smoc*-1 from different mammals

```
Buffalo       ACCGGCCTGGCACCATGCTGCCCGCGCGCTGCGCCGGCCTGCTCACGCCCCACTTGCTGC 95
Cattle        ACCGGCCTGGCACCATGCTGCCCGCGCGCTGCGCCCGCCTGCTCACGCCCCACTTGCTGC 95
Human         CCTGGC-TGGCACCATGCTGCCCGCGCGCTGCGCCCGCCTGCTCACGCCCCACTTGCTGC 299
Chimpanzee    CCTGGT-CGGTACCATGAAGCCCGTGCGCAGCGCCCGCCTGCTCACGCCCCACTTGATGA 299
Rat           --------------ATGCTGCCCGCGCG---CGTCCGTCTGCTCACGCCCCACTTGCTGC 43
Mouse         CTCCGC-TGGCACCATGCTGCCCGCGCG---CGTCCGTCTGCTCACGCCCCACTTGCTGC 278
                            ***  ***** ***   ** *** ****************** **

Buffalo       TGGTGTTAGTGCAGCTGTCCCCGGCTCACGACCACCGCACCACCGGCCCCAGGTTTCTCA 155
Cattle        TGGTGTTAGTGCAGCTGTCCCCGGCTCACGACCACCGCACCACCGGCCCCAGGTTTCTCA 155
Human         TGGTGTTGGTGCAGCTGTCCCCTGCTCGCGGCCACCGCACCACAGGCCCCAGGTTTCTAA 359
Chimpanzee    AGGTGTTTGAGGAGCTGTCCCCAGCTCGGGGCCACCGCACCACATGCCCCAGGTTTCTAA 359
Rat           TCCTGTTGGTGCAGTTGTCCCCGGCCGGCGGCCACCGCACCACCGGCCCCAGGTTTCTAA 103
Mouse         TCGTGTTGGTGCAGCTGTCCCCGGCCGGCGGCCACCGCACCACCGGCCCCAGGTTCCTAA 338
                 **** * **** ******* **    * ************  ********** ** *

Buffalo       TAAGTGACCGTGACCCTCAGTGCAACCTCCACTGCTCCAGGACTCAACCCAAACCTGTCT 215
Cattle        TAAGTGACCGTGACCCTCAGTGCAACCTCCACTGCTCCAGGACTCAACCCAAACCTGTCT 215
Human         TAAGTGACCGTGACCCACAGTGCAACCTCCACTGCTCCAGGACTCAACCCAAACCCATCT 419
Chimpanzee    TAAGTGACCGTGACCCACAGTGCAACCTCCACTGCTCCAGGACTCAACCCAAACCCATCT 419
Rat           TAAGTGACCGTGACCCTCCGTGCAACCCCCACTGCCCCAGGACTCAACCCAAGCCCATCT 163
Mouse         TAAGTGACCGTGACCCTCCGTGCAACCCACACTGTCCCAGGACTCAGCCCAAGCCCATCT 398
              **************** * ********  *****  ********** ***** **  ***

Buffalo       GCGCCTCCGACGGCAGGTCCTACGAGTCCATGTGTGAGTACCAGCGAGCTAAGTGCCGAG 275
Cattle        GCGCCTCCGACGGCAGGTCCTATGAGTCCATGTGTGAGTACCAGCGAGCCAAGTGCCGAG 275
Human         GTGCCTCTGATGGCAGGTCCTACGAGTCCATGTGTGAGTACCAGCGAGCCAAGTGCCGAG 479
Chimpanzee    GTGCCTCTGATGGCAGGTCCTATGAGTCCATGTGTGAGTACCAGCGAGCCAAGTGCCGAG 479
Rat           GCGCGTCTGACGGCAGGTCCTATGAGTCCATGTGTGAGTACCAGAGAGCCAAGTGCCGAG 223
Mouse         GCGCCTCTGACGGCAGGTCCTACGAGTCCATGTGTGAGTACCAGAGAGCCAAGTGCCGAG 458
              * ** ** ** *********** ********************* **** **********

Buffalo       ACCCAACCCTGGCTGTGGCGCATCGAGGCAGATGCAAAGACGCTGGCCAGAGCAAGTGTC 335
Cattle        ACCCAACCCTGGCTGTGGCGCATCGAGGCAGATGCAAAGACGCTGGCCAGAGCAAGTGTC 335
Human         ACCCGACCCTGGGCGTGGTGCATCGAGGTAGATGCAAAGATGCTGGCCAGAGCAAGTGTC 539
Chimpanzee    ACCCGACCCTGGGCGTGGTGCATCGAGGTAGATGCAAAGATGCTGGCCAGAGCAAGTGTC 539
Rat           ACCCTGCCCTGGCCGTGGTCCATCGAGGTAGATGCAAAGATGCTGGCCAAAGCAAGTGTC 283
Mouse         ACCCAGCCCTGGCCGTGGTCCATCGAGGTCGATGCAAAGATGCTGGCCAGAGCAAGTGTC 518
              ****  ******  ****  ********  ********** ******** **********

Buffalo       GCCTGGAGCGGGCTCAGGCCCTGGGGCAAGCCAAGAAGCCCCAGGAGGCGGTGTTTGTCC 395
Cattle        GCCTGGAGCGGGCTCAGGCCCTGGAGCAAGCCAAGAAGCCCCAGGAGGCGGTGTTTGTCC 395
Human         GCCTGGAGCGGGCTCAAGCCCTGGAGCAAGCCAAGAAGCCTCAGGAAGCTGTGTTTGTCC 599
Chimpanzee    GCCTGGAGCGGGCTCAAGCCCTGGAGCAAGCCAAGAAGCCTCAAGAAGCTGTGTTTGTCC 599
Rat           GCCTGGAGAGGGCTCAGGCCTTGGAACAAGCCAAGAAGCCCCAGGAGGCTGTGTTTGTCC 343
Mouse         GCCTGGAGAGGGCTCAGGCCTTGGAACAGGCCAAGAAGCCTCAGGAGGCTGTGTTTGTCC 578
              ******** ******* *** ***  ** *********** ** ** ** **********

Buffalo       CGGAGTGCACCGAGGATGGCTCCTTTACCCAGGTGCAGTGCCATACTTACACCGGGTACT 455
Cattle        CGGAGTGCACCGAGGATGGCTCCTTTACCCAGGTGCAGTGCCATACTTACACCGGGTACT 455
Human         CAGAGTGTGGCGAGGATGGCTCCTTTACCCAGGTGCAGTGCCATACTTACACTGGGTACT 659
Chimpanzee    CAGAGTGTGGCGAGGATGGCTCCTTTACCCAGGTGCAGTGCCATACTTACACTGGGTACT 659
Rat           CAGAGTGTGGCGAGGATGGTTCCTTCACACAGGTGCAGTGTCATACGTACACAGGGTACT 403
Mouse         CAGAGTGTGGCGAGGATGGTTCCTTCACACAGGTGCAGTGCCATACGTACACAGGGTACT 638
              * *****   ********* ***** ** *********** ***** ***** *******

Buffalo       GCTGGTGTGTCACCCCAGACGGGAAGCCCATCAGTGGCTCTTCTGTGCAGAATAAAACTC 515
Cattle        GCTGGTGTGTCACCCCAGACGGGAAGCCCATCAGTGGCTCTTCTGTGCAGAATAAAACTC 515
```

```
Human          GCTGGTGTGTCACCCCGGATGGGAAGCCCATCAGTGGCTCTTCTGTGCAGAATAAAACTC 719
Chimpanzee     GCTGGTGTGTCACCCCGGATGGGAAGCCCATCAGTGGCTCTTCTGTGCAGAATAAAACTC 719
Rat            GCTGGTGTGTCACCCCAGACGGGAAGCCCATCAGTGGCTCGTCCGTGCAGAATAAAACTC 463
Mouse          GCTGGTGTGTCACCCCAGATGGCAAGCCCATCAGTGGTTCTTCCGTGCAGAATAAAACTC 698
               **************** ** ** ************** ** ** ****************

Buffalo        CTGTATGTTCAGGTTCGGTCACCGATAAGCCCGCGAGCCAGGGTAACTCAGGAAGGAAAG 575
Cattle         CTGTATGTTCAGGTTCGGTCACCGATAAGCCTGCGAGCCAGGGTAACTCAGGAAGGAAAG 575
Human          CTGTATGTTCAGGTTCAGTCACCGACAAGCCCTTGAGCCAGGGTAACTCAGGAAGGAAAG 779
Chimpanzee     CTGTATGTTCAGGTTCAGTCACCGACAAGCCCTTGAGCCAGGGTAACTCAGGAAGGAAAG 779
Rat            CTGTATGTTCAGGTCCAGTCACTGACAAGCCCTTGAGCCAGGGTAACTCAGGAAGGAAAG 523
Mouse          CTGTATGTTCAGGTCCAGTTACCGACAAGCCCTTGAGCCAGGGTAATTCAGGAAGAAAAG 758
               ************** * ** ** ** *****   ************ ******** ****

Buffalo        ATGACGGGTCTAAGCCGACACCCACGATGGAGACCCAGCCGGTGTTCGATGGAGACGAAA 635
Cattle         ATGATGGGTCTAAGCCGACACCCACGATGGAGACCCAGCCGGTGTTCGATGGAGACGAAA 635
Human          ATGACGGGTCTAAGCCGACACCCACGATGGAGACCCAGCCGGTGTTCGATGGAGATGAAA 839
Chimpanzee     ATGACGGGTCTAAGCCGACACCCACGATGGAGACCCAGCCGGTGTTCGATGGAGATGAAA 839
Rat            ATGATGGCTCTAAACCCACGCCCACGATGGAGACCCAGCCTGTGTTCGATGGAGATGAAA 583
Mouse          ATGATGGCTCTAAACCCACGCCCACGATGGAGACCCAACCTGTGTTCGATGGAGATGAAA 818
               **** ** ***** ** ** ***************** ** ************** ****

Buffalo        TCACAGCTCCCACTCTCTGGATTAAGCACTTGGTAATCAAGGACTCCAAACTGAACAACA 695
Cattle         TCACAGCTCCCACTCTCTGGATTAAGCACTTGGTAATCAAGGACTCCAAACTGAACAACA 695
Human          TCACAGCCCCAACTCTATGGATTAAACACTTGGTGATCAAGGACTCCAAACTGAACAACA 899
Chimpanzee     TCACAGCCCCAACTCTATGGATTAAACACTTGGTGATCAAGGACTCCAAACTGAACAACA 899
Rat            TCACAGCCCCTACCTTATGGATTAAGCACTTGGTAATCAAAGACTCCAAATTGAATAACA 643
Mouse          TCACAGCCCCCACCTTATGGATTAAGCACTTGGTAATCAAAGACTCCAAGTTGAATAACA 878
               ******* ** **  * ******** ******** ***** ********  **** ****

Buffalo        CCAACATAAGAAATTCAGAGAAAGTTCACTCGTGTGACCAGGAGAGACAGAGCGCCCTGG 755
Cattle         CCAACATAAGAAATTCAGAGAAAGTTCACTCGTGTGACCAGGAGAGACAGAGCGCCCTGG 755
Human          CCAACATAAGAAATTCAGAGAAAGTCTATTCGTGTGACCAGGAGAGGCAGAGTGCCCTGG 959
Chimpanzee     CCAACATAAGAAATTCAGAGAAAGTCTACTCGTGTGACCAGGAGAGGCAGAGTGCCCTGG 959
Rat            CCAATGTAAGAAATTCAGAGAAAGTCCATTCTTGTGACCAGGAGAGACAGAGCGCCCTGG 703
Mouse          CCAACGTAAGAAATTCAGAGAAAGTCCATTCCTGTGACCAGGAAAGACAGAGTGCCCTGG 938
               ****  *******************  * ** *********** ** ***** *******

Buffalo        AAGAGGCCCGGCAGAACCCCCGCGAGGGCATTGTGATCCCCGAGTGTGCTCCTGGGGGGC 815
Cattle         AAGAGGCCCGGCAGAACCCCCGCGAGGGCATTGTGATCCCTGAGTGTGCTCCTGGGGGAC 815
Human          AAGAGGCCCAGCAGAATCCCCGTGAGGGTATTGTCATCCCTGAATGTGCCCCTGGGGGAC 1019
Chimpanzee     AAGAGGCCCGGCAGAATCCCCGTGAGGGTATTGTCATTCCTGAATGTGCCCCTGGGGGAC 1019
Rat            AAGAGGCCCGGCAGAATCCTCGAGAGGGCATTGTGATCCCCGAGTGTGCTCCTGGTGGGC 763
Mouse          AAGAGGCCCGGCAGAATCCCCGAGAGGGCATTGTGATCCCCGAGTGTGCTCCTGGTGGGC 998
               ********* ****** ** ** ***** ***** ** ** ** ***** ***** ** *

Buffalo        TCTATAAACCAGTGCAGTGCCACCAGTCCACTGGCTACTGCTGGTGTGTGCTGGTGGACA 875
Cattle         TCTATAAACCAGTGCAGTGCCACCAGTCCACTGGCTACTGCTGGTGTGTGCTGGTGGACA 875
Human          TCTATAAGCCAGTGCAATGCCACCAGTCCACTGGCTACTGCTGGTGTGTGCTGGTGGACA 1079
Chimpanzee     TCTATAAGCCAGTGCAATGCCACCAGTCCACTGGCTACTGCTGGTGTGTGCTGGTGGACA 1079
Rat            TCTATAAGCCGGTGCAATGCCACCAATCCACGGGCTACTGCTGGTGTGTCCTAGTGGACA 823
Mouse          TCTATAAGCCGGTGCAATGCCACCAATCCACAGGCTACTGTTGGTGCGTCCTAGTAGACA 1058
               ******* ** ***** ******** ***** ******** ***** ** ** ** ****

Buffalo        CTGGGCGTCCGCTGCCGGGGACCTCCACACGCTATGTGATGCCCAGTTGTGAGAGTGATG 935
Cattle         CTGGGCGTCCGCTGCCGGGGACCTCCACACGCTACGTGATGCCCAGTTGTGAGAGTGATG 935
Human          CAGGGCGCCCGCTGCCTGGGACCTCCACACGCTACGTGATGCCCAGTTGTGAGAGCGACG 1139
Chimpanzee     CAGGGCGCCCGCTGCCTGGGACCTCCACACGCTACGTGATGCCCAGTTGTGAGAGCGACG 1139
Rat            CAGGACGCCCATTGCCGGGGACTTCCACACGCTATGTGATGCCAAGTTGTGAGAGTGACG 883
Mouse          CAGGGCGCCCATTGCCGGGGACTTCCACACGCTATGTGATGCCAAGTTGCGAGAGTGACG 1118
               * ** ** **  **** ***** *********** ******** ***** ***** ** *

Buffalo        CCAGGGCTAAGAGTGCGGAGGTGGAGGACCCCTTCAAGGACAGGGAGCTGCCAGGCTGTC 995
```

```
Cattle       CCAGGGCTAAGAGTGCGGAGGTGGAGGACCCCTTCAAGGACAGGGAGCTGCCAGGCTGTC 995
Human        CCAGGGCCAAGACTACAGAGGCGGATGACCCCTTCAAGGACAGGGAGCTACCAGGCTGTC 1199
Chimpanzee   CCAGGGCCAAGACTACAGAGGCGGATGACCCCTTCAAGGACAGGGAGCTACCAGGCTGTC 1199
Rat          CCAGAGCCAAGAGTGTAGAGGTGGATGACCCTTTCAAGGACAGGGAGTTACCAGGCTGTC 943
Mouse        CCAGAGCCAAGAGTGTAGAGGCCGATGACCCTTTCAAGGACAGGGAGTTGCCAGGTTGTC 1178
             **** ** **** *   ****  ** ***** *************** * ***** ****

Buffalo      CAGAAGGGAAGAAACTGGAATTTATCACCAGCCTTCTGGACGCCCTCACCACGGACATGG 1055
Cattle       CAGAAGGGAAGAAACTGGAATTTATCACCAGCCTACTGGACGCCCTCACCACTGACATGG 1055
Human        CAGAAGGGAAGAAAATGGAGTTTATCACCAGCCTACTGGATGCTCTCACCACTGACATGG 1259
Chimpanzee   CAGAAGGGAAGAAAATGGAGTTTATCACCAGCCTACTGGATGCTCTCACCACTGACATGG 1259
Rat          CTGAAGGGAAGAAGATGGAATTTATCACCAGCCTGCTGGATGCCCTCACCACAGACATGG 1003
Mouse        CTGAAGGGAAGAAGATGGAATTTATTACCAGCCTGCTGGATGCCCTCACCACAGACATGG 1238
             * ***********  **** ***** ******** ***** ** ******** *******

Buffalo      TGCAGGCCATTAACTCAGCAGCGCCCACTGGAGGTGGGAGGTTCTCGGAGCCAGACCCCA 1115
Cattle       TGCAGGCCATTAACTCAGCAGCGCCCACTGGAGGTGGGAGGTTCTCGGAGCCAGACCCCA 1115
Human        TTCAGGCCATTAACTCAGCAGCGCCCACTGGAGGTGGGAGGTTCTCAGAGCCAGACCCCA 1319
Chimpanzee   TTCAGGCCATTAACTCAGCAGCGCCCACTGGAGGTGGGAGGTTCTCAGAGCCAGACCCCA 1319
Rat          TTCAGGCCATTAACTCAGCAGCGCCCACTGGAGGTGGGAGGTTCTCAGAGCCAGACCCCA 1063
Mouse        TTCAGGCCATTAACTCAGCAGCGCCCACTGGAGGTGGGAGGTTCTCAGAGCCAGACCCCA 1298
             * ******************************************** *************

Buffalo      GCCACACCCTGGAGGAGCGCGTGGTGCACTGGTATTTCAGCCAGCTGGACAGCAACAGCA 1175
Cattle       GCCACACCCTGGAGGAGCGCGTGGTGCACTGGTATTTCAGCCAGCTGGACAGCAACAGCA 1175
Human        GCCACACCCTGGAGGAGCGGGTAGTGCACTGGTATTTCAGCCAGCTGGACAGCAATAGCA 1379
Chimpanzee   GCCACACCCTGGAGGAGCGGGTAGTGCACTGGTATTTCAGCCAGCTGGACAGCAATAGCA 1379
Rat          GCCACACCCTGGAGGAGCGGGTGGCACACTGGTACTTCAGCCAGCTGGATAGCAACAGCA 1123
Mouse        GCCACACCCTGGAGGAGCGAGTGGCACACTGGTACTTCAGCCAGCTGGATAGCAACAGCA 1358
             ******************* ** *  ******** ************** ***** ****

Buffalo      GCAGCGACATCAACAAGCGCGAGATGAAGCCCTTCAAGCGCTATGTGAAGAAGAAAGCCA 1235
Cattle       GCAGCGACATCAACAAGCGCGAGATGAAGCCCTTCAAGCGCTACGTAAAGAAGAAAGCCA 1235
Human        GCAACGACATTAACAAGCGGGAGATGAAGCCCTTCAAGCGCTACGTGAAGAAGAAAGCCA 1439
Chimpanzee   GCAACGACATTAACAAGCGGGAGATGAAGCCCTTCAAGCGCTATGTGAAGAAGAAAGCCA 1439
Rat          GTGATGACATTAACAAGCGGGAGATGAAACCGTTCAAGCGCTATGTGAAGAAGAAAGCCA 1183
Mouse        GCGATGACATTAACAAGCGGGAGATGAAGCCGTTCAAGCGCTATGTAAAGAAGAAAGCCA 1418
             *    ***** ******** ******** ** *********** ** *************

Buffalo      AGCCCAAGAAATGTGCCCGGCGTTTCACTGACTACTGTGACCTGAACAAGGACAAGGTCA 1295
Cattle       AGCCCAAGAAATGTGCCCGGCGTTTCACTGACTACTGTGACCTGAACAAGGACAAGGTCA 1295
Human        AGCCCAAGAAATGTGCCCGGCGTTTCACCGACTACTGTGACCTGAACAAAGACAAGGTCA 1499
Chimpanzee   AGCCCAAGAAATGTGCCCGGCGTTTCACCGACTACTGTGACCTGAACAAAGACAAGGTCA 1499
Rat          AGCCCAAGAAGTGCGCCCGGCGCTTCACCGACTACTGTGACCTGAACAAGGATAAGGCCA 1243
Mouse        AGCCCAAGAAATGTGCCCGGCGCTTCACCGACTACTGCGACCTGAACAAGGATAAGGTCA 1478
             ********** ** ******** ***** ******** *********** ** **** **

Buffalo      TCTCACTGCCCGAGCTGAAGGGCTGCCTGGGTGTTAGCAAAGAAGTAG------------ 1343
Cattle       TCTCACTGCCCGAGCTGAAGGGCTGCCTGGGTGTTAGCAAAGAAG--------------- 1340
Human        TTTCACTGCCTGAGCTGAAGGGCTGCCTGGGTGTTAGCAAAGAAG--------------- 1544
Chimpanzee   TTTCACTGCCTGAGCTGAAGGGCTGCCTGGGTGTTAGCAAAGAAG--------------- 1544
Rat          TCTCGCTGCCTGAGCTGAAGGGCTGCCTGGGTGTTAGCAAAGAAGGTGGTAGCCTTGGCA 1303
Mouse        TCTCACTGCCTGAGCTGAAGGGCTGCCTGGGTGTTAGCAAAGAAGGTGGTAGCCTTGGCA 1538
             * ** ***** **********************************

Buffalo      ------------------------------------------GACGCCTCGTCTAAGGAG 1361
Cattle       ------------------------------------------GACGCCTCGTCTAA---- 1354
Human        ------------------------------------------GACGCCTCGTCTAAGGAG 1562
Chimpanzee   ------------------------------------------GACGCCTCGTCTAAGGAG 1562
Rat          GCTTCCCTCAGGGAAAACGAGCAGGCACAAATCCATTCATTGGACGTCTCGTCTAA---- 1359
Mouse        GCTTCCCTCAGGGAAAACGAGCAGGCACAAACCCGTTCATCGGACGCCTTGTCTAAAGAG 1598
                                                       **** ** ******
```

```
Buffalo      CAGAAAGC-CAAAGGGCAGGTGGAGAGACCAGGGAGGCAGGATGGATCATCAGA------ 1414
Cattle       ------------------------------------------------------------
Human        CAGAAAAC-CCAAGGGCAGGTGGAGAGTCCAGGGAGGCAGGATGGATCACCAGACACCTA 1621
Chimpanzee   CAGAAAAC-CCAAGGGCAGGTGGAGAGTCCAGGGAGGCAGGATGGACCACCAGACACCTA 1621
Rat          ------------------------------------------------------------
Mouse        TGGAAAAAGCAAAAGGCCCATGGAAACTCCGAGGAGACAGGACTGACCGTCAGACACCTA 1658
```
