# Supplementary material for: Characterization of Smoc-1 uncovers two transcript variants showing differential tissue and age specific expression in Bubalus bubalis
Source: BMC Genomics. 2007 Nov 28;8:436. doi: 10.1186/1471-2164-8-436 (PMC2235864; doi:10.1186/1471-2164-8-436)
Supplement: Additional file 4 — Evolutionary conservation of Smoc-1 across the species. Cross hybridization of buffalo Smoc-1 with genomic DNA from different species (A), Phylogenetic tree based on sequence alignment of Smoc-1 gene(s) from different species (B) and neighbor joining tree based on BLAST result showing homology across the species with their accession numbers (C). Note that this gene is phylogentically conserved across the species. [file 1471-2164-8-436-S4.pdf]

(A)

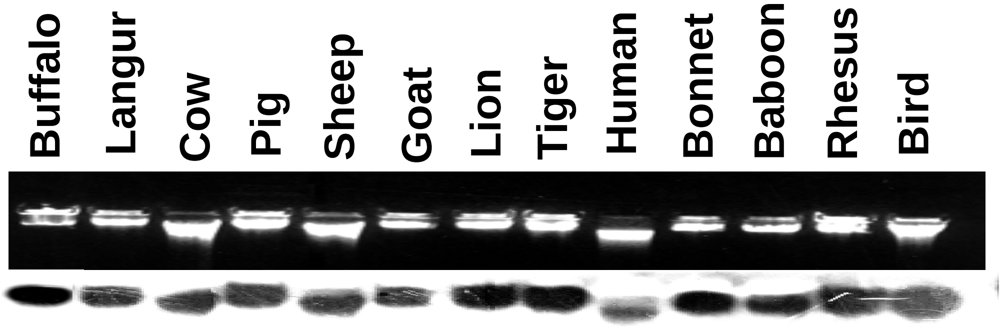

(B)

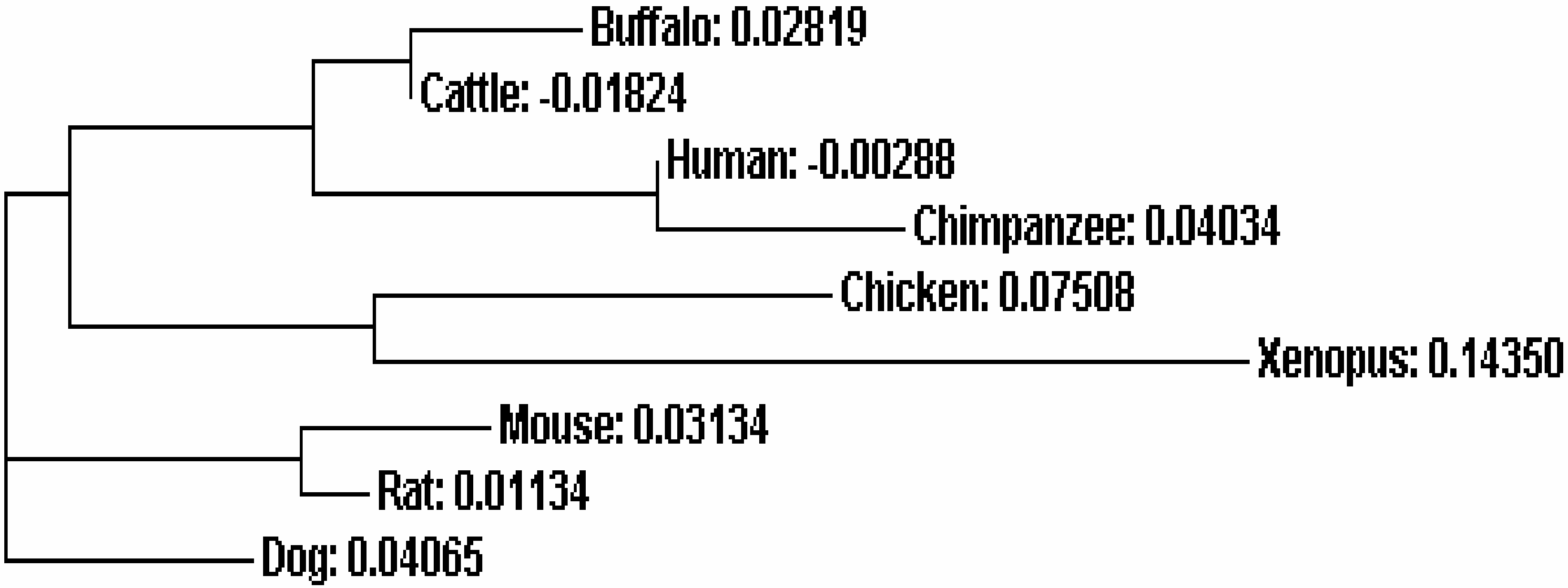

Additional file 4

Contd/-

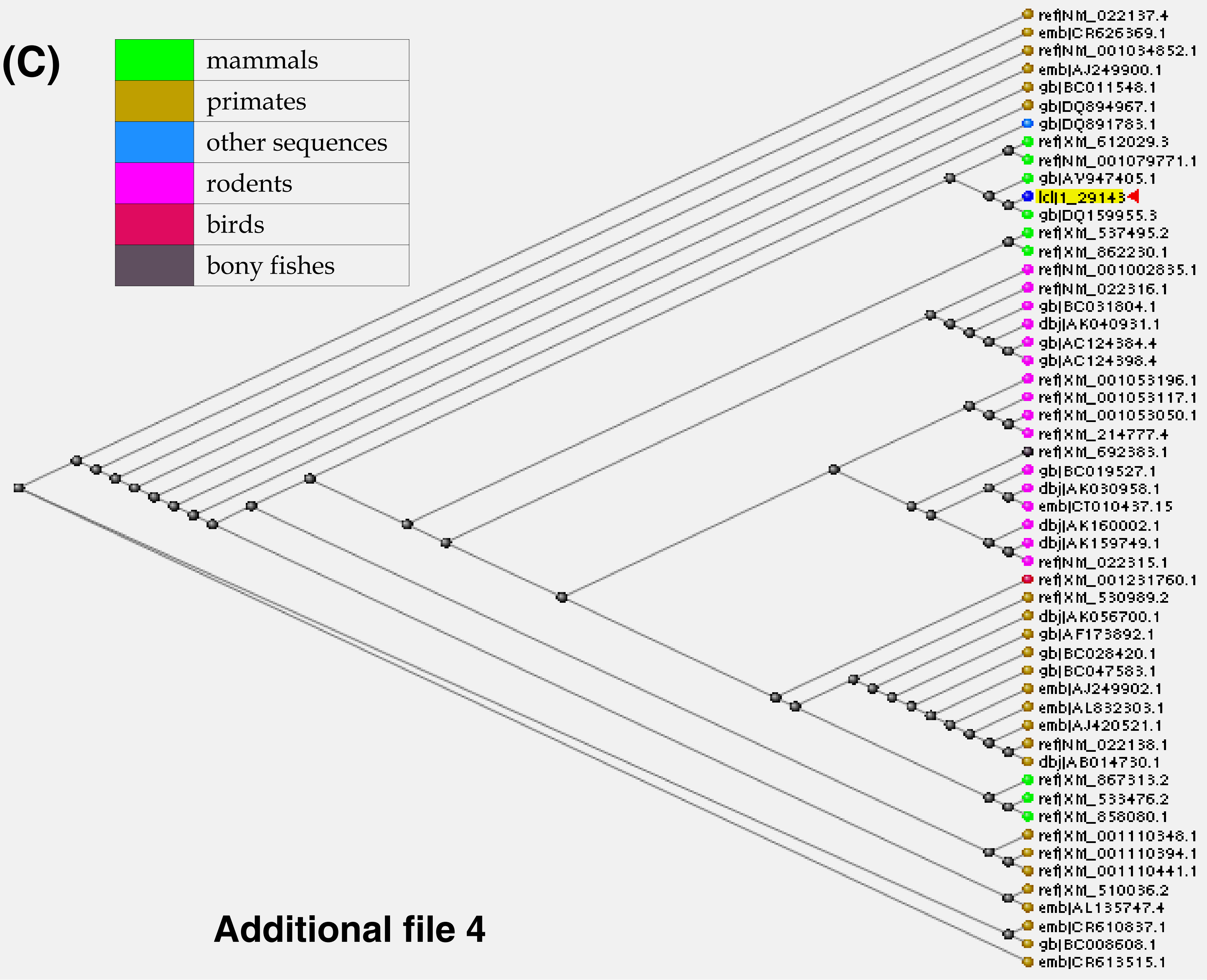

Additional file 4
