# Supplementary figures and images for: Characterization of Smoc-1 uncovers two transcript variants showing differential tissue and age specific expression in Bubalus bubalis
Source: BMC Genomics. 2007 Nov 28;8:436. doi: 10.1186/1471-2164-8-436 (PMC2235864; doi:10.1186/1471-2164-8-436)

Delta Rn vs Cycle

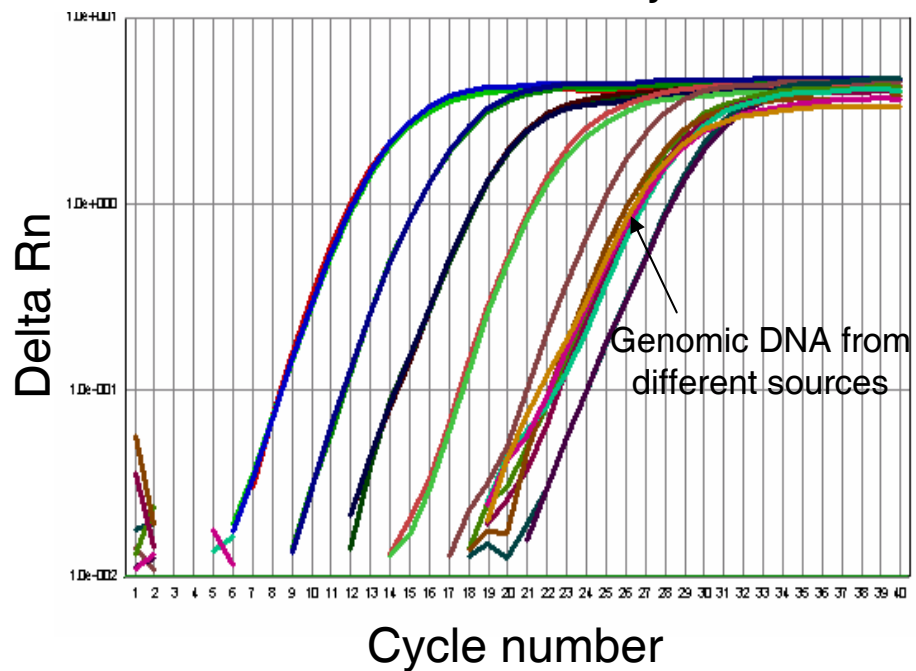

(A)

Standard curve

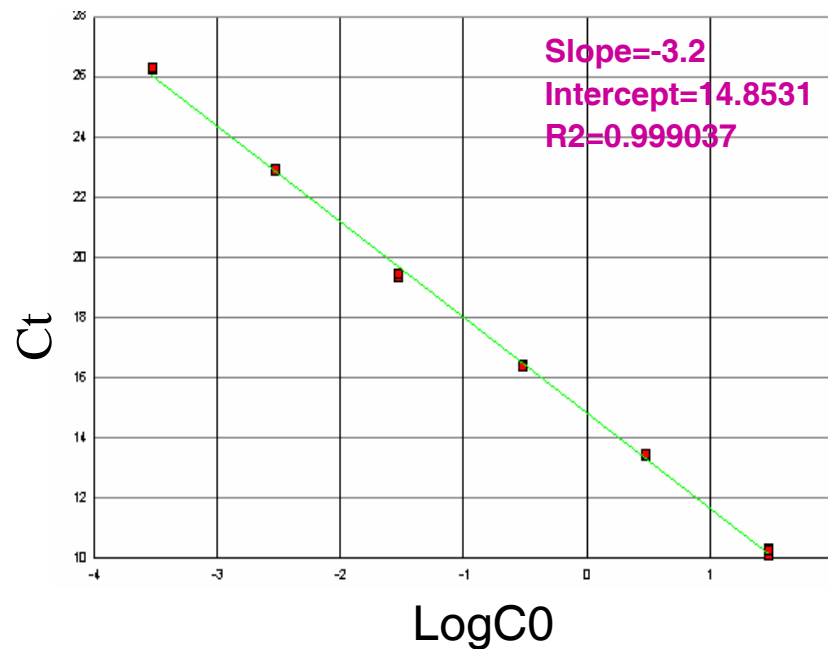

(B)

Additional file 6

Supplement: Additional file 6 — Copy number calculation of Smoc-1 gene. Real Time PCR amplification plot based on ten fold dilution series of FSmoc-1 recombinant plasmid (A). Genomic DNA from blood of male/female buffalo and semen samples used as template (A) to obtain a standard curve using SYBR Green assay (B) which detected the single copy status of this gene. The value of R2, slope and intercept are given in the standard curve. [file 1471-2164-8-436-S6.pdf]

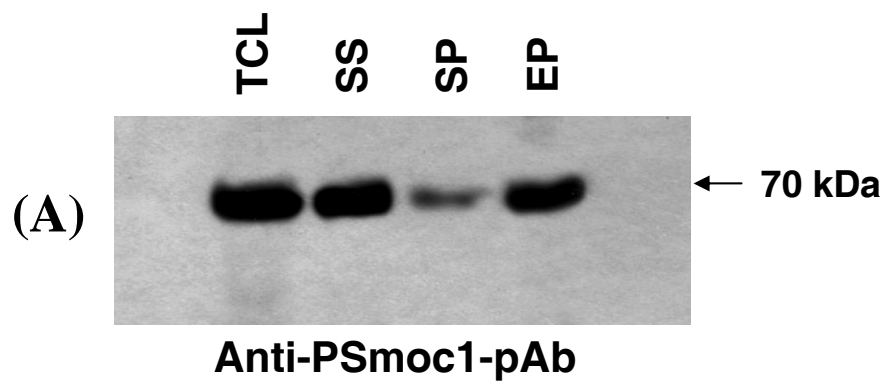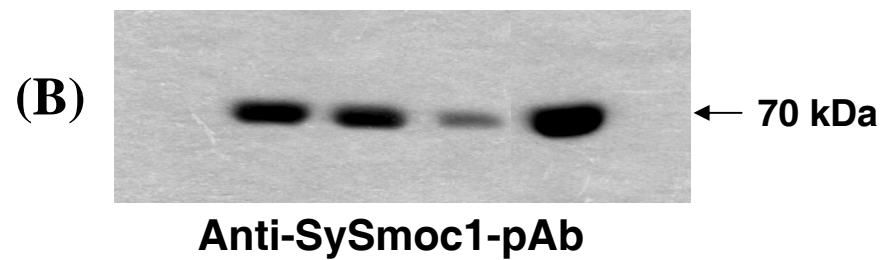

**Additional file 7**

Supplement: Additional file 7 — Western blot using anti-Smoc-1 antibodies. Anti-PSmoc1-pAb specifically generated against GST-Smoc1 recombinant protein showed ~70 kDa protein in western blotting (A). The same results were observed using Anti-SySmoc-1-pAb generated against the synthesized amino acids specific to Smoc-1 unique domain (B). TCL denotes total cell lysate; SS, sonicated supernatant; SP, sonicated pellet and EP, eluted protein. [file 1471-2164-8-436-S7.pdf]
