# Supplementary material for: Characterization of Smoc-1 uncovers two transcript variants showing differential tissue and age specific expression in Bubalus bubalis
Source: BMC Genomics. 2007 Nov 28;8:436. doi: 10.1186/1471-2164-8-436 (PMC2235864; doi:10.1186/1471-2164-8-436)
Supplement: Additional file 8 — Details of primers used for analysis of Smoc-1. List of primers used for amplification of full length Smoc-1CDS, Its relative expression and copy number calculation. The size of oligos, their annealing temperature and corresponding product size of the respective clones have been given in the table. [file 1471-2164-8-436-S8.pdf]

Additional file 8: Details of Primers used for amplification of full length *Smoc-1 CDS*, Its relative expression and copy number calculation

S.N.	Clone ID	Primer code	Sequence(5'-3')	Mers	Annealing temp.	Product size
**For full length CDS amplification**						
1.	Clone II	JS275	5' ATGACTGTGTCCCCTGACCGCAGC 3'	24	65	1414
2.		JS276	5' TCTGATGATCCATCCTGCCTCCCTGGT 3'	27	67	
3.	Clone III	JS985	5' TGTGACCTGAACAAGGACAAGGT 3'	23	62	1009
4.		JS986	5' TTCTGTGCTTCTACCTCCACTGC 3'	23	62	
5.	Clone IV	JS990	5' AACTTTCTTCACGGAGGTGCTTC 3'	23	61	1030
6.		JS991	5' AATTGCCCTAAGGATTTGCGTTA 3'	23	61	
7.	Clone V	JS996	5' AAATGTAATATCTGAGCAGTGGAGGT 3'	26	60	1000
8.		JS997	5' TATAAAACAAGCTACAAACGGTCTCC 3'	26	60	
9.	Clone VII	JS977	5' GTTTCACTGACTACTGTGACCTGAAC 3'	26	65	506
10.	Clone VIII	JS997	5' TATAAAACAAGCTACAAACGGTCTCC 3'	26	60	1018
11.	Clone VI	J5UTR	5' CAGCAGTACCCGGTGTAAGTATG 3'	23	63	649
**For copy number calculation and relative expression**						
10.	Variants-01+-02	JSR1015	F 5' CGCGTGGTGCACTGGTATT 3'	19	60	72
11.		JSR1016	R 5' CTTCATCTCGCGCTTGTTGA 3'	19	60	
12.		JSR1017	F 5' ATCAACAAGCGCGAGATGAAG 3'	21	60	74
13.		JSR1018	R 5' CGCCGGGCACATTTCTT 3'	17	60	
14.	Variant-01	JSR1033	F 5' AGGGAGGGTGGGCAGTTTT 3'	19	60	74
15.		JSR1034	R 5' TGCGGCCAGATTTCCAAA 3'	18	60	
