# Supplementary material for: Characterization of Smoc-1 uncovers two transcript variants showing differential tissue and age specific expression in Bubalus bubalis
Source: BMC Genomics. 2007 Nov 28;8:436. doi: 10.1186/1471-2164-8-436 (PMC2235864; doi:10.1186/1471-2164-8-436)
Supplement: Additional file 5 — Secondary structure of Smoc-1 protein from different species. Predicted secondary structures of Smoc-1 protein in different species. The replacement of helix formed by 8 residues with the beta-sheets, insertion of helices and the minor alterations throughout the protein are shown in red. Changes similar to cattle or human and the ones similar to rat/mouse or chimpanzee are shown in red and blue, respectively. The additional coils and helices in mouse and rat are due to bigger coding frame of Smoc-1 in these species. [file 1471-2164-8-436-S5.pdf]

Additional file 5: Secondary structure of Smoc-1 from different species

```
Buffalo       CCHHHHHHHHHHHEEEEEEEECCCCCCCCCCCCHH--HHCCCCCCCCCCCCCCCCCCCCC 57
Cattle        CCHHHHHHHHHHHHHHHHHEECCCCCCCCCCCC-----ECCCCCCCCCCCCCCCCCCCCC 54
Chimpanzee    CCHHHHHHHHHHHHHHHHHH--CCCCCCCCCCC----CCCCCCCCCCCCCCCCCCCCCCC 54
Mouse         CCCCHHHHHHHHHHHHHHHH--CCCCCCCCCCCEEEECCCCCCCCCCCCCCCCCCCCCCC 58
Human         CCHHHHHHHHHHHHHHHHHH--CCCCCCCCCCC----CCCCCCCCCCCCCCCCCCCCCCC 54
Rat           CCHHHHHHHHHHHHHHHHHH--CCCCCCCCCCCE--EEECCCCCCCCCCCCCCCCCCEEC 56
              **  ********          ***********      ******************  *

Buffalo       CCCCCEEEE---CCCCCCCCCCCCCCCCCCCCCCCCCCCHHHHHHHHHHHHCCCCCCCEE 114
Cattle        CCCCCECC----CCCCCCCCCCCCCCCECCCCCCCCCCCHHHHHHHHHHHHCCCCCCCCC 114
Chimpanzee    CCCCCCCC-----CCCCCCCCCC----CCCCCCCCCCCCHHHHHHHHHHHHCCCCCCCEE 114
Mouse         CCCCCCCC-----CCCCCCCCCCCEEECCCCCCCCCCCCHCHHCCCCCHHHCCCCCCCCC 113
Human         CCCCCCCCECEEECCCCCCCCCCCCCCCCCCCCCCCCCCHHHHHHHHHHHHCCCCCCCCC 114
Rat           CCCCEECCH--HHHHHCCCCCCCCEEECCCCCCCCCCCCHHHHHHHHHHHHCCCCCCCC- 113
              ****  **        *******     ************ **     **********

Buffalo       CCCCCCCCCCEEEECCCCCCEEEEECCCCCCCCCCCCCCCCCCCCCCCCCCCCCCCCCCC 174
Cattle        CCCCCCCCCEEEEECCCCCCEEEEECCCCCCCCCCCCCCCCCCCCCCCCCCCCCCCCCCC 174
Chimpanzee    CCCCCCCCCCEECCCCCCCCEEEECCCCCCCCCCCCCCCCCCCCCCCCCCCCCCCCCCCC 174
Mouse         CCCCCCCCCCEEEECCCCCCEEEEECCCCCCCCCCCCCCCCCCCCCCCCCCCCCCCCCCC 173
Human         CCCCCCCCCCECEECCCCCCEEEEECCCCCCCCCCCCCCCCCCCCCCCCCCCCCCCCCCC 174
Rat           CCCCCCCCCEEECECCCCCCEEEEECCCCCCCCCCCCCCCCCCCCCCCCCCCCCCCCCCC 173
              ********* *   ****** ***************************************

Buffalo       CCCCCCCCCCCCCCCCCCCCCCCCCCCCCCCCCCCCCCCCCCCCCCCCCCCC-------- 226
Cattle        CCCCCCCCCCCCCCCCCCCCCCCCCCCCCCCCCCCCCCCCCCCCCCCCCCCC-------- 226
Chimpanzee    CCCCCCCCCCCCCCCCCCCCCCCCCCCCCEECCCCCCCCCCCCCCCCCCCCC-------- 226
Mouse         CCCCCCCCCCCCCCCCCCCCCCCCCCCCCCCCCCCCCCCCCCCCCCCCCCCCCCCCCCCC 233
Human         CCCCCCCCCCCCCCCCCCCCCCCCCCCCHHCCCCCCCCCCCCCCCCCCCCCC-------- 226
Rat           CCCCCCCCCCCCCCCCCCCCCCCCCCCCCCCCCCCCCCCCCCCCCCCCCCCC-------- 225
              ****************************   *********************

Buffalo       ---HHHHHHHHHHHHCCCCCCCEEEECCCCCCCEEEEEECCCCCEEEEEEHHHECECCCC 283
Cattle        ---HHHHHHHHHHHHCCCCCCCCEECCCCCCCCEEECECCCCCCEEEEEECCCCCECCCC 283
Chimpanzee    ---HHHHHHHHHHHHCCCCCCCEEECCCCCCCCCECCCCCCCCCEEEEEECCCCCCCCCC 283
Mouse         CCHHHHHHHHHHHHHCCCCCCCCEECCCCCCCCCECCCCCCCCCEEEEEECCCCCCCCCC 293
Human         ---HHHHHHHHHHHHCCCCCCCCEECCCCCCCCCECEECCCCCCEEEEEECCCCCCCCCC 283
Rat           ---HHHHHHHHHHHHCCCCCCCEEECCCCCCCCECCCCCCCCCCEEEEEECCCCCCCCCC 282
                 ******************* **********     ***************** ****

Buffalo       CCCCCCCCCCCCCCCCCCCCCCCCCCCCCCCCCCCHHHHHHHHHHHHCCCEEEEECCCCC 343
Cattle        CCCCCCCCCCCCCCCCCCCCCCCCCCCCCCCCCCCCHHHHHHHHHHHCCCEEEEECCCCC 343
Chimpanzee    CCCCCCCCCCCCCCCCCCCCCCCCCCCCCCCCCCCHHHHHHHHHHHHCCCEEEEECCCCC 343
Mouse         CCCCCCCCCCCCCCCCCCCCCCCCCCCCCCCCCCCHHHHHHHHHHHHCCCEEEECCCCCC 353
Human         CCCCCCCCCCCCCCCCCCCCCCCCCCCCCCCCCCCCHHHHHHHHHHHCCCEEEEECCCCC 343
Rat           CCCCCCCCCCCCCCCCCCCCCCCCCCCCCCCCCCCCHHHHHHHHHHHHHHCCCCCCCCCC 342
              *********************************** ************  *    *****

Buffalo       CCCCCCCCCCCCCCCCCCEEEEEEHHHECCCCCCCCCHHHHHHHHHHHHHHHHHHHHHC 402
Cattle        CCCCCCCCCCCCCCCCCCEEEEEEHHHECCCCCCCCCHHHHHHHHHHHHHHHHHHHHHC 402
Chimpanzee    CCCCCCCCCCCCCCCCHHCEEEEEEEECCCCCCCCCCHHHHHHHHHHHHHHHHHHHHHC 402
Mouse         CCCCCCCCCCCCCCCCHHCEEEEEEEEECCCCCCCCCHHHHHHHHHHHHHHHHHHHHHC 412
Human         CCCCCCCCCCCCCCCCCHCEEEEEEEECCCCCCCCCCHHHHHHHHHHHHHHHHHHHHHC 402
Rat           CCCCCCECCCCCCCCCCCCEEEEEHHECCCCCCCCCCHHHHHHHHHHHHHHHHHHHHHC 401
              ****** *********   *****... *******************************

Buffalo       CCCEEECCCCCCEECHHHHHHHCCCCCCCCCCC------------------ 435
Cattle        CCCEEECCCCCCEECHHHHHHHCCCCCCCCCC------------------- 434
Chimpanzee    CCCEEECCCCCCEECHHHHHHHCCCCCCCCCC------------------- 434
Mouse         CCCEEECCCCCCEECHHHHHHCCCCCCCCCCCCCCCCCCCCCCCCCCHHHCC 463
Human         CCCEEECCCCCCEECHHHHHHHCCCCCCCCCC------------------- 434
Rat           CCCEEECCCCCCEECHHHHHHHCCCCCCCCCCCCCCCCCCCCCCCCCCHHHCC 452
              ********************* **********
```
